# Whole-genome analysis reveals the contribution of non-coding de novo transposon insertions to autism spectrum disorder

**DOI:** 10.1186/s13100-021-00256-w

**Published:** 2021-11-27

**Authors:** Rebeca Borges-Monroy, Chong Chu, Caroline Dias, Jaejoon Choi, Soohyun Lee, Yue Gao, Taehwan Shin, Peter J. Park, Christopher A. Walsh, Eunjung Alice Lee

**Affiliations:** 1 Division of Genetics and Genomics, Manton Center for Orphan Disease, Boston Children’s Hospital, Boston, MA USA; 2 Broad Institute of MIT and Harvard, Cambridge, MA 02142 USA; 3 Department of Biomedical Informatics, Harvard Medical School, Boston, MA USA; 4 Division of Developmental Medicine, Boston Children’s Hospital, Harvard Medical School, Boston, MA USA; 5 Department of Genetics, Harvard Medical School, MA Boston, USA; 6 Department of Pediatrics, Harvard Medical School, MA Boston, USA; 7 Department of Neurology, Harvard Medical School, Boston, MA USA; 8 Howard Hughes Medical Institute, Boston Children’s Hospital, Boston, MA USA

**Keywords:** Transposable elements, Retrotransposons, Autism spectrum disorder, de novo insertions, Polymorphic insertions, de novo rates, Alu, SVA, LINE-1, Neurobiology

## Abstract

**Background:**

Retrotransposons have been implicated as causes of Mendelian disease, but their role in autism spectrum disorder (ASD) has not been systematically defined, because they are only called with adequate sensitivity from whole genome sequencing (WGS) data and a large enough cohort for this analysis has only recently become available.

**Results:**

We analyzed WGS data from a cohort of 2288 ASD families from the Simons Simplex Collection by establishing a scalable computational pipeline for retrotransposon insertion detection. We report 86,154 polymorphic retrotransposon insertions—including > 60% not previously reported—and 158 de novo retrotransposition events. The overall burden of de novo events was similar between ASD individuals and unaffected siblings, with 1 de novo insertion per 29, 117, and 206 births for Alu, L1, and SVA respectively, and 1 de novo insertion per 21 births total. However, ASD cases showed more de novo L1 insertions than expected in ASD genes. Additionally, we observed exonic insertions in loss-of-function intolerant genes, including a likely pathogenic exonic insertion in *CSDE1*, only in ASD individuals.

**Conclusions:**

These findings suggest a modest, but important, impact of intronic and exonic retrotransposon insertions in ASD, show the importance of WGS for their analysis, and highlight the utility of specific bioinformatic tools for high-throughput detection of retrotransposon insertions.

**Supplementary Information:**

The online version contains supplementary material available at 10.1186/s13100-021-00256-w.

## Background

Retrotransposons contribute to genomic and transcriptomic variability in humans and cause a variety of human diseases [1]. Retrotransposons are a class of mobile DNA elements that can copy themselves into RNA and insert themselves into new regions of the genome. This retrotransposition event is estimated to occur in one out of 20-40, 63-212, and 63-916 live births for Alu, LINE-1 (L1), and SVA elements respectively [2–4]. Transposable element insertions (TEIs) in both exons and non-coding regions can cause diseases by various mechanisms, including disrupting coding sequences, causing deletions, and altering RNA splicing, which can cause frameshifts and loss of function (LoF) [1]. To date, there are more than 100 cases of TEIs causing diseases [1], including de novo insertions in developmental disorders [5]. A landmark study identified a deep intronic SVA insertion causing exon-trapping in a child with Batten disease, resulting in the development of a personalized antisense-oligonucleotide drug to fix the splicing defect [6]. Thus, the identification of TEs is important for increasing genetic diagnoses but also creates the promise of developing novel therapeutics for specific mutant alleles.

**Fig. 1:**
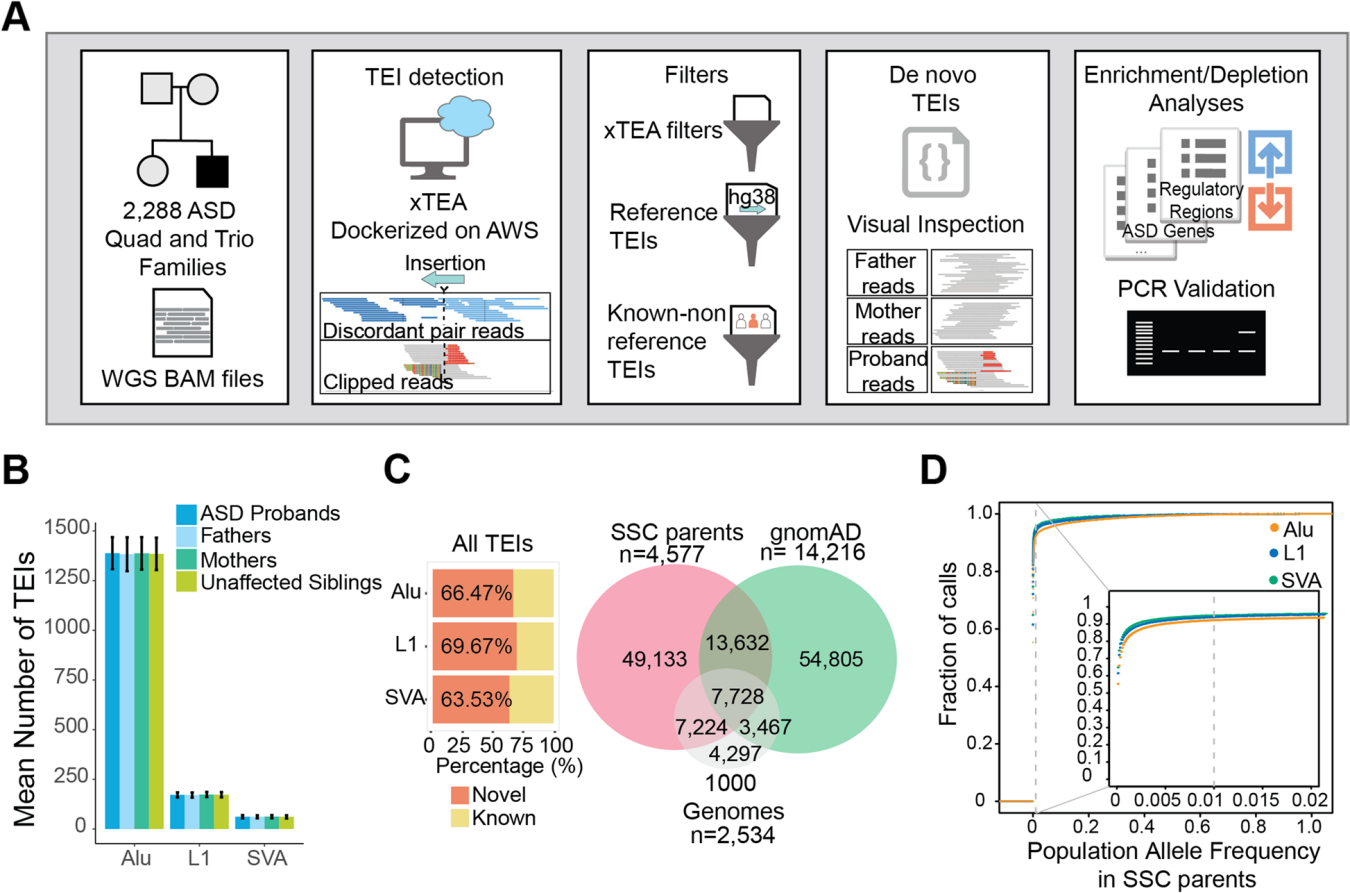
Detection of transposable element insertions (TEIs) in the SSC cohort. **A** Pipeline and analysis overview. Quad and trio bam files were analyzed for TEIs using a dockerized version of *xTea* on the cloud in Amazon Web Services (AWS). Candidate TE insertions were filtered using xTea filters, and filters for regions of the genome with reference and known non-reference TEIs for a high confidence set. A custom pipeline for detection of de novo insertions was used, and candidates were manually inspected on the Integrative Genomics Viewer. Enrichment or depletion of TEIs in ASD genes, high pLI genes, genomic regions, and regulatory regions in fetal brain development was tested by simulation analyses. A subset of candidates was validated by full-length PCR. **B** Mean number of TEIs detected in the SSC cohort with standard deviation. **C** Percentage of insertions in the SSC cohort that were not found in previous studies (novel) or overlap with TEIs from previous analyses (known) for all TEIs including those in parents and children (left) and Venn diagram showing overlap with other large cohort studies for TEIs detected in unrelated parental samples in our cohort (right). **D** Cumulative fraction of TEIs in unrelated parental samples which are found at a certain population allele frequency (PAF) within the SSC cohort. 94% L1, 92% Alu, and 95% SVA insertions show < 1% PAF

Autism Spectrum Disorder (ASD) is a heterogeneous developmental disorder characterized by communication deficits, impaired social interactions, and repetitive behaviors [7]. Although about 17-50% of the overall heritability of ASD reflects common variation at a population level, rare inherited and de novo copy-number variations and single nucleotide variations confer high risk to developing ASD, and drive ASD risk when present in individual children [8]. These rare variants are enriched in simplex families, where both parents are unaffected, with de novo copy-number variations and single nucleotide variations contributing to 30% of cases in the Simons Simplex Cohort (SSC) [9]. Although recent ASD studies have included TEIs [10–12], the smaller sample size and the low rates of de novo TEIs limited their analyses leaving the role of de novo TEIs in both exons and introns in ASD largely unknown. In this study, we sought to define the role of TEIs in ASD by analyzing the largest cohort of 2288 simplex families for de novo TEIs at whole genome resolution (Fig. 1A).

## Results

### Most polymorphic insertions are rare and novel

We developed and implemented *xTea* [13], a scalable algorithm for detecting TEIs in whole-genome sequencing (WGS) data and demonstrated that the version of this tool used in our study has a high sensitivity, specificity, and comparable performance to MELT [14], the algorithm used to detect TEIs in gnomAD, as well as a better performance than Mobster [15] (Additional file 1: Fig. S1). Using *xTea*, we detected a total of 86,154 unique polymorphic TEIs (68,643 Alu, 12,076 L1, and 5435 SVA) in the entire cohort (parents and children) (Additional file 1: Fig. S2A and Table S1). Each genome carried 1618 polymorphic TEIs on average (1385 Alu, 172 L1, and 61 SVA) comparable with previous analyses [16, 17], and the numbers were consistent across different family members (Fig. 1B and Additional file 1: Fig. S2A). We detected more Alu TEIs in African Americans, suggesting that TEI diversity is different in distinct populations (F(7) = 970.8, *p* < 2e-16, one-way ANOVA) (Additional file 1: Fig. S3). 74% of the overall TEIs detected (50,507 Alu, 9247 L1, 4273 SVA) were observed in either more than one individual in this cohort (71%; 48,189 Alu, 8821 L1, 4021 SVA) or in previous studies (33%; 23,018 Alu, 3663 L1, 1982 SVA) (Additional file 1: Fig. S2B), suggesting that most of these calls are bona fide. However, more than 60% of calls were novel and had not been detected before in gnomAD [18] or the 1000 genomes cohort [14] (Fig. 1C and Additional file 1: Fig. S4). In 4577 unrelated parental samples in our cohort, we detected 77,717 TEIs (dbVar “nstd203”), compared to the 79,632 insertions detected from 54,805 individuals in the gnomAD-SV cohort [18]. The majority of novel TEIs had the expected target-site duplication (TSD) size, and SVA and L1 novel TEIs display a similar TSD size distribution to known non-reference (KNR) TEIs (Additional file 1: Fig. S5A). The TSD size distribution of novel Alu TEIs with sufficient clipped and discordant read support on both breakpoints of the insertion, a polyA tail, and a TSD also resembles the TSD size distribution of KNR TEIs (Additional file 1: Fig. S5B). The high performance, especially high specificity (> 75% for Alu, L1, and SVA at ~40X sequencing coverage) of *xTea* (Additional file 1: Fig. S1) suggests that the majority of these novel TEIs are high confidence insertions. Additionally, insertions in our cohort had a higher overlap with previously published insertions from 2534 individuals in the 1000 genomes cohort [14] (Fig. 1C). The majority of parental TEIs were rare, for example, > 92% of TEIs having < 1% population allele frequency (PAF) within the analyzed cohort (Fig. 1D and Additional file 1: Fig. S6), which is similar to previous findings for structural variants [18].

### ASD cases have more de novo insertions in ASD genes than expected

**Fig. 2:**
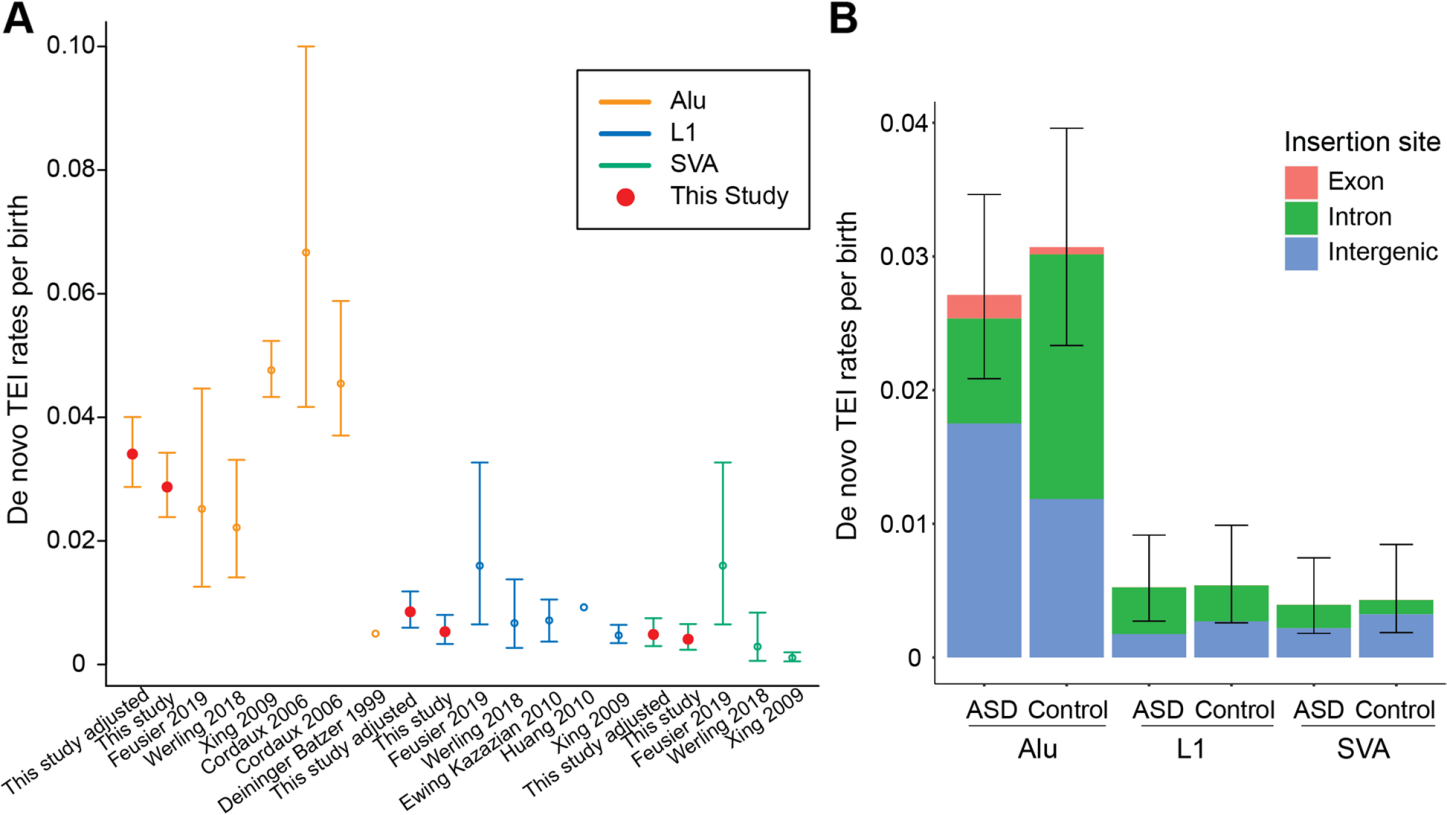
Rates of de novo TEIs. **A** Combined rates of de novo TEIs per birth for ASD and controls compared to previous studies. The adjusted rate in our study accounts for lower sensitivity for detecting TEIs in short-read Illumina data compared to long-read sequencing data. **B** Rates of de novo TEIs per birth in probands with ASD and unaffected siblings (controls)

We identified 158 de novo TEIs from all children (Additional file 2: Table S2). Previous studies have generally reported de novo TEI rates based on the number of insertions found in their cohort without accounting for detection sensitivity [4, 12]. Multiple factors, including filtered regions, low sensitivity of the algorithm being used, or false negatives due to the sequencing methodology, result in an underestimate of true de novo rates. For example, TEI detection in Illumina short-read sequencing data is less sensitive than in long-read data, particularly for L1 TEIs [19, 20]. Therefore, we adjusted the observed de novo rates to account for sensitivity loss and to obtain precise estimates. We obtained adjusted de novo rates of 1 in 29 births for Alu (95% CI 25-35), 1 in 117 births for L1 (95% CI 85-168), and 1 in 206 births for SVA (95% CI 134-336) (Fig. 2A and Additional file 1: Table S3).

We detected 62 de novo Alu insertions in ASD (*N* = 2286) and 57 in controls (*N* = 1857), 12 de novo L1 insertions in ASD (N = 2286) and 10 in controls (*N* = 1856), and 9 de novo SVA insertions in ASD (*N* = 2288) and 8 in controls (*N* = 1860) (Additional file 2: Table S2). We did not detect a difference in total de novo TEIs in ASD versus unaffected siblings (Fig. 2B) but unexpectedly observed a higher rate of intronic Alu insertions in controls (*p* = 0.003, two-sided Fisher’s Exact Test) (Fig. 2B). On the other hand, we observed a trend towards more exonic and intergenic Alu insertions in ASD than controls though not significant (*p* = 0.388 for exonic insertions, *p* = 0.157 for intergenic insertions, two-sided Fisher’s Exact Test) (Fig. 2B) which leads to similar overall rates for de novo Alu insertions.

**Fig. 3:**
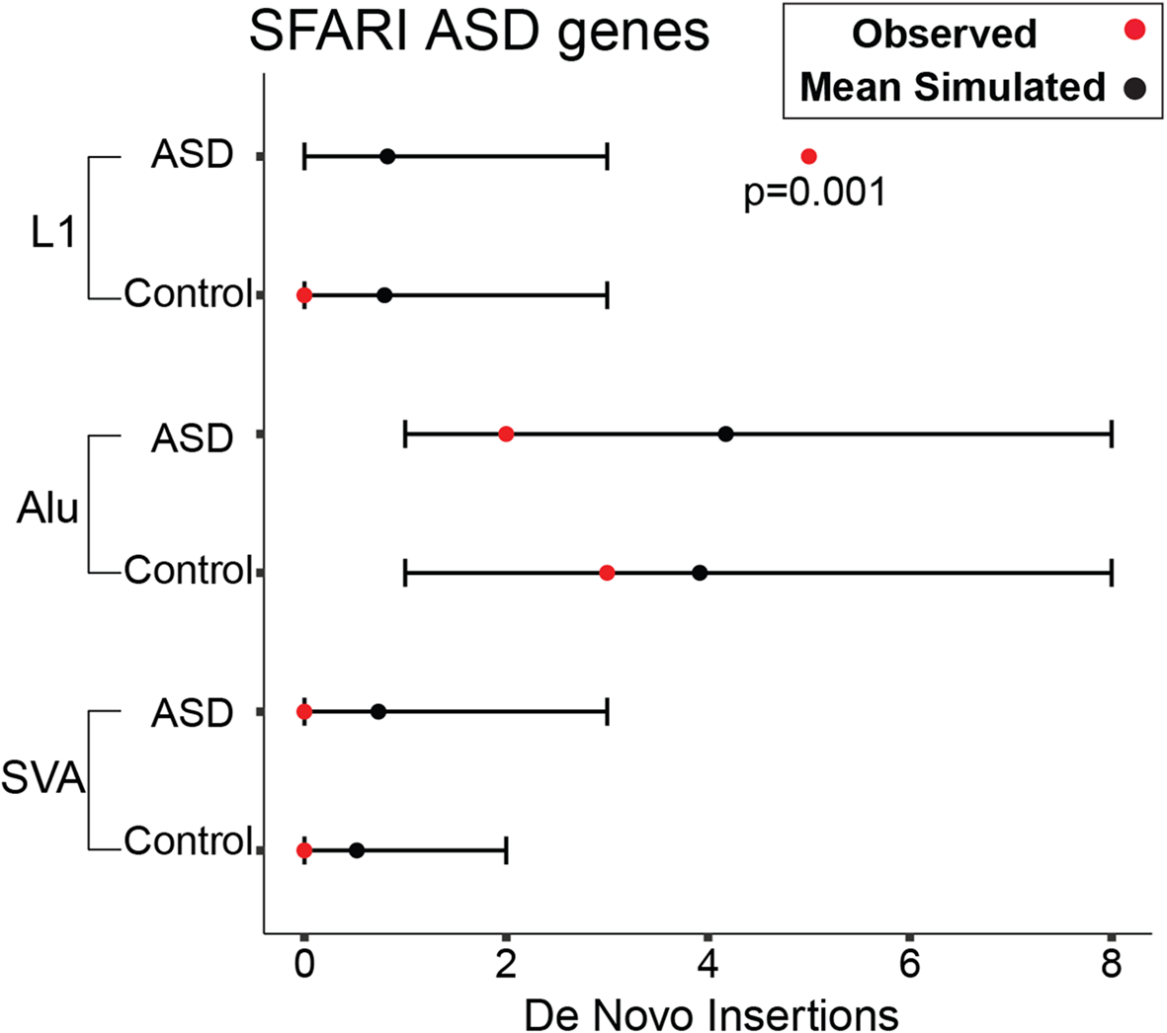
Enrichment of de novo TEIs in SFARI ASD genes. Observed numbers of de novo TEIs in a list of complied ASD genes are marked by red dots. Black dots and lines represent mean numbers and 95% confidence intervals of expected TEIs based on 10,000 random simulations, respectively. More de novo L1 insertions in ASD genes than expected are observed in cases only

We detected de novo intronic L1 insertions in syndromic ASD genes curated by Simons Foundation Autism Research Initiative (SFARI) [21] only in ASD and not in controls, and the rate in ASD was higher than expected (empirical two-sided *p*-value using 10,000 permutation runs, *p* = 0.001, q-value = 0.03) (Fig. 3) (Table 1). We also observed a trend for more de novo intronic L1 insertions in genes with high pLI scores indicating a high probability of loss of function intolerance [36] in ASD than expected (empirical two-sided *p*-value, *p* = 0.02, q-value > 0.05) (Additional file 1: Fig. S7). This approach is limited to observing an enrichment or depletion compared to a fully random model of TEIs, and we cannot account for factors that might influence the location of de novo insertions such as GC content and epigenetic context. However, these trends were also confirmed when simulating random insertions that consider the L1 endonuclease cleavage site [37, 38] (Additional file 1: Fig. S8). We identified de novo exonic insertions in genes with a high probability of LoF intolerance or haploinsufficiency (pLI ≥ 0.9) [36] only in affected individuals (Table 1 and Additional file 2: Table S2), including an exonic insertion in *CSDE1*, a gene recently implicated in patients with ASD and neurodevelopmental disabilities [22]. There is a large overlap between SFARI genes and high pLI genes with de novo L1 insertions in cases; 80% (4/5) of SFARI genes with L1 insertions in ASD are also high pLI genes, suggesting that the de novo events can disrupt the haploinsufficient ASD genes and contribute to ASD risk (Table 1).

**Table 1:** Select de novo insertions in ASD and high pLI genes in affected individuals

Proband ID	TE Type	Gene	SFARI Classification^**a**^	pLI	Genic Region	Validation Status	Observed Phenotype	Previous Neurodevelopmental Phenotype associated with gene	Reference
11,859.p1	Alu	CSDE1	No	1	Exon	Validated	ASD, language delay, ID, macrocephaly, history of vision correction, normal EEG at 4 years	LGD variants associated with ASD, developmental delay, ID, seizures, macrocephaly, ADHD, anxiety, ocular abnormalities	Guo et al. 2019 [22]
14,565.p1	Alu	KBTBD6	No	0.935	Exon	Validated	ASD, macrocephaly, uncoordinated, normal IQ, BMI Z-score − 3.91		
12,548.p1	Alu	APPBP2	No	0.999	Intron	Validated	ASD, normal IQ, macrocephaly		
12,748.p1	Alu	SYT1	Syndromic	0.837	Intron	Validated	ASD, normal IQ, uncoordinated	Developmental delays, autistic features, hypotonia, ocular abnormalities, hyperkinetic movements associated with de novo missense variation	Baker et al., 2018 [23]
13,931.p1	Alu	OTUD7A	Suggestive evidence	0.975	Intron	Validated	ASD, borderline IQ, normal EEG, and brain imaging	Neurodevelopmental phenotype of ASD, developmental delay, ID, seizures associated with 15q13.3 microdeletion syndrome	Yin et al. 2018 [24], Uddin et al. 2018 [25]
13,107.p1	Alu	TOX3	No	0.994	Intron	Validated	ASD, normal IQ		
14,315.p1	Alu	JAZF1	No	0.958	Intron	Validated	ASD, borderline verbal IQ, normal nonverbal IQ, normal EEG		
11,196.p1	L1	SRGAP3	Minimal Evidence	1	Intron	Validated	ASD, above average IQ, no history of seizures, heart problems reported	Case report of translocation breakpoint at loci posited to be LoF associated with hypotonia and severe ID	Endris et al. 2002 [26]
13,684.p1	L1	HCN1	Syndromic	0.953	Intron	NA	ASD, Tourette syndrome, above average IQ, GI problems, uncoordinated	Missense variation associated with a syndrome of seizures, intellectual disability, and autistic features, gene also implicated in Tourette syndrome, role in striatal neuronal function and enteric nervous system	Nava et al. 2014 [27], Tsetsos et al. [28] 2021
14,080.p1	L1	DAB1	Hypothesized	0.981	Intron	Validated	ASD, uncoordinated, GI problems	ASD, GI problems, schizophrenia, spinocerebellar ataxia-37 associated with non-coding nucleotide repeats	Corral-Juan et al. 2018 [29], Nawa et al. 2020 [30]
14,282.p1	L1	DPYD	Suggestive evidence	0	Intron	Not in LCL DNA, predicted blood mosaic	ASD, normal IQ	ASD, ID	Carter et al. 2011 [31], Willemsen et al. 2011 [32]
11,234.p1	L1	NOTCH2	No	1	Intron	NA	ASD, above average IQ		
13,451.p1	L1	DPP10	Suggestive evidence	1	Intron	NA	ASD, borderline IQ	ASD	Marshall et al. 2008 [33]
14,404.p1	SVA	GRAMD1B	No	0.985	Intron	NA, predicted blood mosaic	ASD, non-verbal, IQ in profound intellectual disability range, macrocephaly	Autosomal recessive intellectual disability	Santos-Cortez et al. 2018 [34]
14,523.p1	SVA	ACACA	No	1	Intron	NA	ASD, above average IQ, macrocephaly	Acetyl-CoA carboxylase deficiency	Blom et al. 1981 [35]

A subset of de novo TEIs observed in individuals with ASD in genes relevant to ASD or with a high probability of being loss-of-function intolerant (pLI > 0.9). ^a^SFARI annotations were obtained in 2019. *LCL* Lymphoblastoid cell line, *LGD* Likely gene disrupting, *ID* Intellectual disability, *LoF* Loss of function, *ADHD* Attention-deficit hyperactivity disorder, *GI* Gastrointestinal.

### De novo insertion size distribution resembles polymorphic insertions

Since paternal and maternal age presents a risk to ASD [39], we tested whether there was a difference in parental age at birth in children with and without de novo TEIs. We found a modest, but not significant, increase in paternal age for children with de novo TEIs compared to those without de novo TEIs (M = 33.94, SD = 5.63 vs. M = 33.29, SD = 4.71; t(163.42) = 1.4452, *p* = 0.1503) as well as increase in maternal age (M = 31.62, SD = 4.92 vs. M = 31.12, SD = 4.92; t(163.75) = 1.29, *p* = 0.198) (Additional file 1: Fig. S9). We also estimated the insertion size of polymorphic and de novo TEIs by mapping insertion-supporting reads from *xTea* output to TE consensus sequences and obtaining the minimum and maximum mapping coordinates. The distribution of polymorphic L1 insertion size closely resembles previously published data [14] (Additional file 1: Fig. S10A). Overall, de novo TEIs showed similar size distributions to polymorphic TEIs but had different patterns from somatic TEIs, which showed more severe 5′ truncation [17] (Additional file 1: Fig. S10B).

### De novo insertions in ASD show an enrichment trend in regulatory regions of the fetal brain

**Fig. 4:**
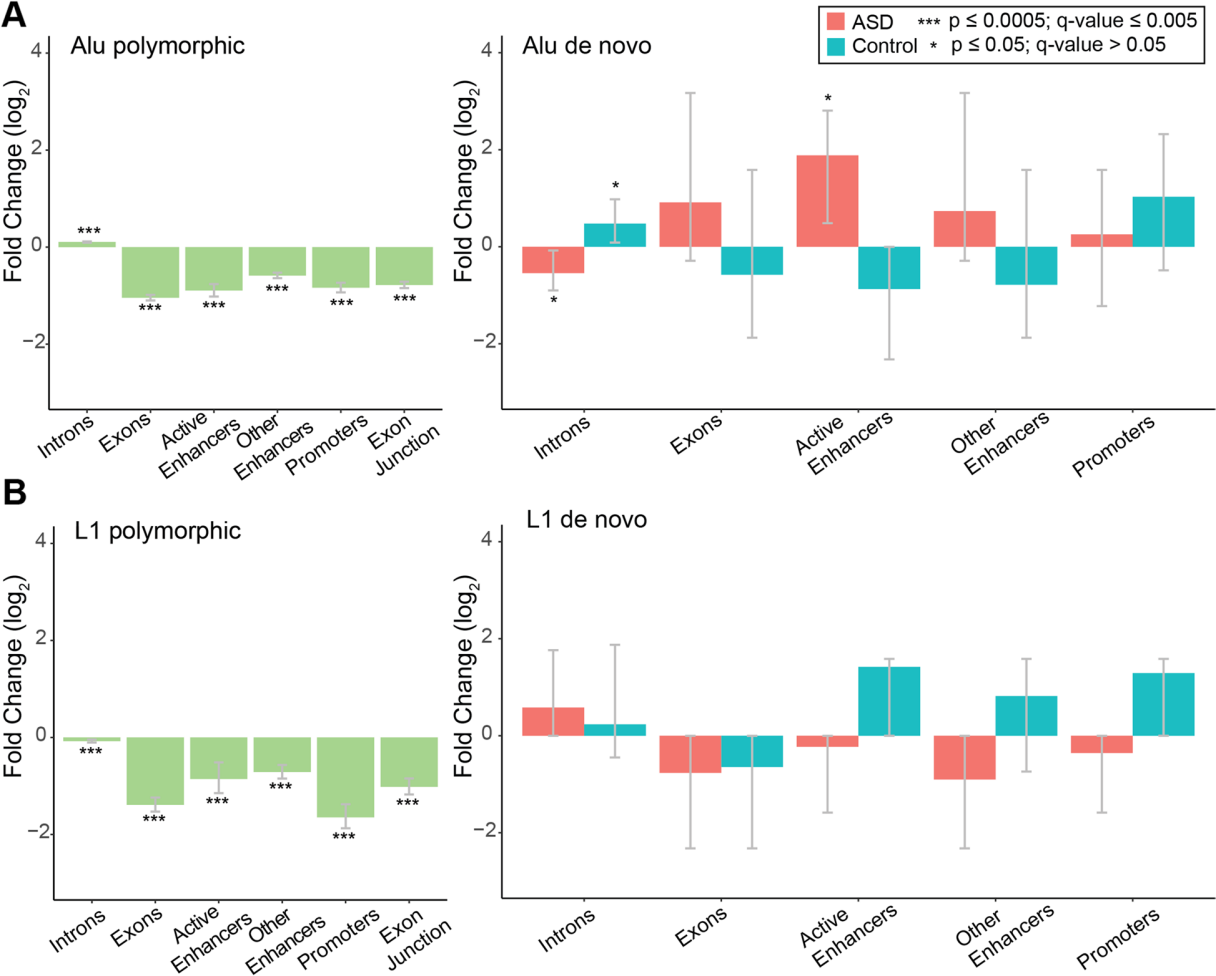
Genomic distribution of polymorphic and de novo TEIs. **A** 10,000 random simulations were performed for both polymorphic and de novo TEIs based on the observed rates. Log_2_ fold change of observed compared to expected counts in different genomic regions are shown for coding and gene regulatory regions. 95% confidence intervals were estimated based on the empirical distribution of the random simulations. Polymorphic TEIs from parental individuals are depleted in exons and regulatory regions in the developing fetal brain. De novo Alu (**A**) and L1 insertions (**B**) do not show this depletion compared to 10,000 random simulations. Two-sided empirical *p*-values and Benjamini–Yekutieli q-values based on multiple correction of all enrichment and depletions performed are represented

Some genes with de novo TEIs in ASD are highly expressed in the brain at all stages of development (Additional file 1: Table S4). We found an enrichment of de novo TEIs in ASD in genes upregulated in the prefrontal cortex, although this was not significant after multiple test correction (*p*-value = 0.0017, Benjamini-Hochberg q-value = 0.07), whereas no such enrichment was detected in controls. Additionally, we found that genes with de novo TEIs were enriched for calcium-dependent phospholipid-binding in ASD (adjusted *p*-value = 0.034) but did not find enrichment for any Gene Ontology terms in controls. Several de novo TEIs were also observed in regions with enhancer and promoter chromatin marks in fetal brain development (Additional file 1: Table S5). Thus, we evaluated the enrichment of polymorphic and de novo TEIs in different genomic and epigenomic regions using the Roadmap Epigenomics 25-state model [40]. Polymorphic L1 and Alu insertions were depleted in exons, enhancers, and promoters (Fig. 4; two-sided empirical *p* < 0.0005, Benjamini–Yekutieli q-value< 0.0043 for each category) whereas SVAs did not show a significant depletion in those regions likely due to the limited number of insertions (Additional file 1: Fig. S11 and Table S6). De novo TEIs overall showed patterns within the expected ranges in most regions, however, we observed a trend for more de novo Alu insertions in active enhancer regions in the fetal brain in ASD than expected but not in controls (two-sided empirical *p* = 0.018, Benjamini–Yekutieli q-value = 0.3). This trend was also observed when considering the L1 endonuclease cleavage site preference in the background model for expected TEIs (Additional file 1: Fig. S12). This suggests the intriguing possibility that Alu insertions in neural enhancers might be a rare cause of ASD, though larger samples sizes are needed to test this.

### PCR validations confirm ASD relevant retrotransposon insertions

**Fig. 5:**
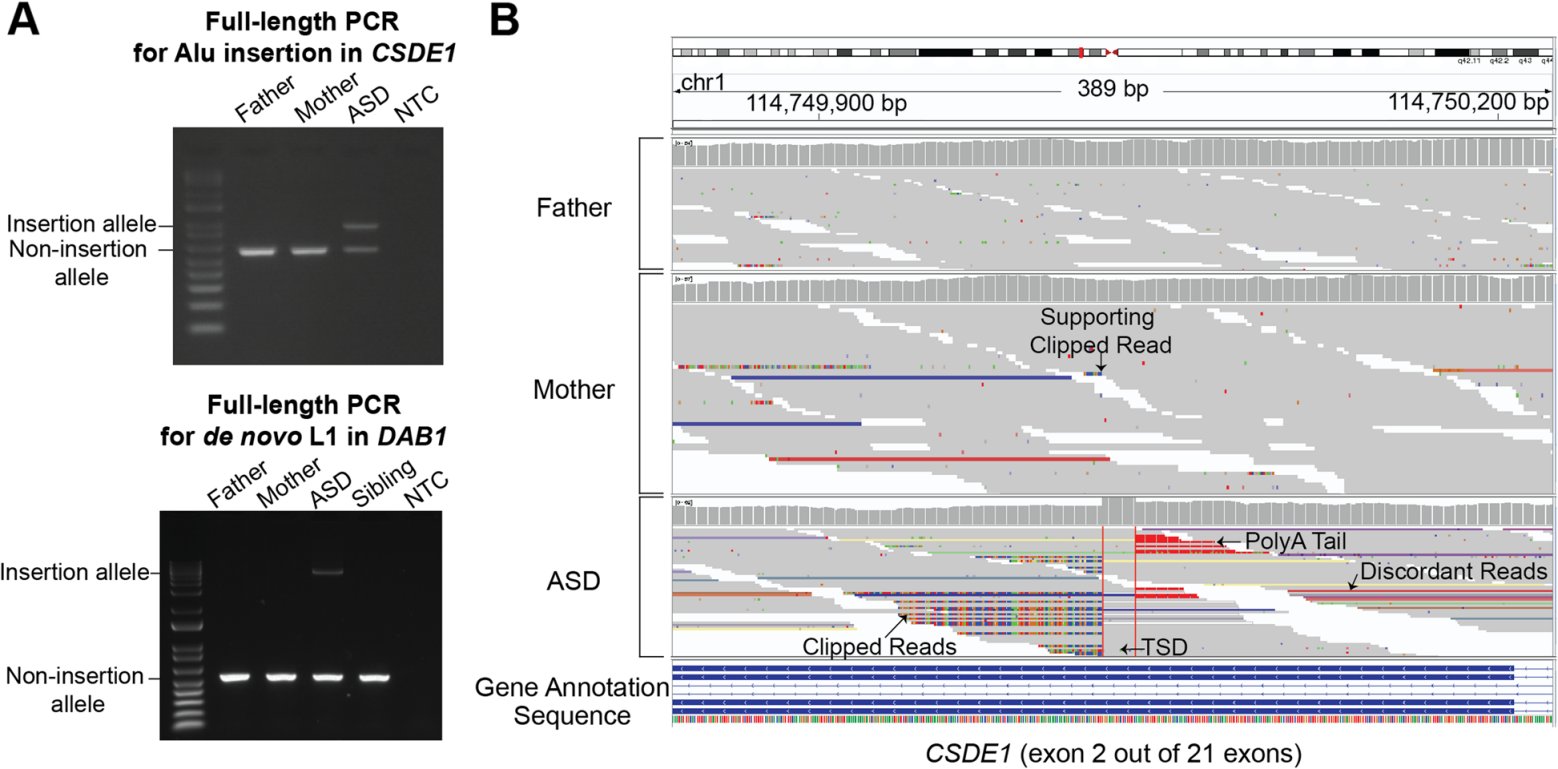
Full-length PCR validations and visual inspection. **A** Full-length PCR validation of the Alu insertion in *CSDE1* and of the de novo L1 insertion in *DAB1* in ASD cases. In lymphoblastoid cell line DNA, we validated the insertions in the ASD proband only. NTC: non-template control. **B** Integrative Genomics Viewer image at the insertion site in gene *CSDE1* in an ASD case. For each individual, the sequencing coverage (top) and sequencing reads (bottom) are shown. The insertion shows the canonical signatures of target-primed reverse transcription (TPRT)-mediated retrotransposition: 15 bp target site duplication (TSD) between the two insertion breakpoints, a poly-A tail, supporting clipped reads, and discordant reads with mates mapping to the consensus Alu sequence. The mother has one small clipped read sequence at the breakpoint which has the same sequence as in the proband, suggesting that the insertion could be mosaic at a low allele frequency in the mother’s blood

We selected de novo L1 and Alu insertions from both cases and controls in a subset of ASD and high pLI genes as well as in randomly selected genes for full-length PCR validation (Additional file 3: Table S7). We validated 22 of 23 (96%) Alu insertions and 6/7 (86%) L1 insertions, achieving a high validation rate of 93% (28/30). Validated insertions include a full-length de novo intronic L1 insertion in *DAB1,* a gene with a high probability of being loss-of-function intolerant (pLI = 0.981) [36] and a hypothesized ASD gene [21, 30] implicated in regulating neuronal migration in development via the Reelin pathway in an isoform dependent manner [41]. We additionally validated an exonic Alu insertion in ASD gene *CSDE1* [22] in an ASD proband (Fig. 5A)*.* Our manual IGV inspection identified a single supporting clipped read at the breakpoint (Fig. 5B) in the mother, suggesting that the exonic Alu insertion in *CSDE1* could be potentially mosaic at a low allelic fraction in the mother’s blood, though low-level contamination from the proband’s DNA cannot be completely ruled out. This insertion was fully validated in lymphoblastoid cell line (LCL) DNA in the individual with ASD and was absent in the mother, but LCLs might be expected to be limited in validating low-level mosaic variants (Fig. 5A).

## Discussion

The detection of TEIs in genome sequencing data requires specific pipelines, given their repetitive nature and short read length. These variants have previously been excluded from most routine genetic diagnoses and studies, including for ASD. Furthermore, accurate estimation of de novo TEIs in healthy individuals is important to understand the contribution of de novo TEIs in disease cohorts. Initial methods to determine de novo rates of TEIs relied on indirect methods which compared two reference genomes, making assumptions regarding the time to the most recent common ancestor between human reference genomes [3] and human-chimpanzee divergence time [2]. To directly determine de novo retrotransposition rates, large cohorts are necessary given the infrequency of these events. More recent studies using short-read sequencing technologies have included fewer than 1000 families each, leading to uncertainties in estimates, especially for SVA insertions [4, 10, 11]. They have also not accounted for the lower sensitivity of detection on TEIs using short-read sequencing [12]. Compared to 1 in 20 [2] or 1 in 21 [3] Alu insertions per birth by earlier studies using evolutionary and mutational based methods, our estimate of 1 in 29 births is lower but within the range from more recent work using family genome sequencing data of 1 in 39.7 births (95% CI 22.4–79.4) [4] (Fig. 2A). L1 rates observed here of 1 in 117 births are also within the ranges observed previously of 1 in 63 births (95% CI 30.6–153.8) [4] and 1 in 149.2 (95% CI 72.5-370.4) [10] but higher than the Xing et al. 2009 rate of 1 in 212 births (95% CI 156-289) [3] (Fig. 2A). Our SVA de novo rates of 1 in 206 births are much higher than the Xing et al. 2009 rate of 1 in 916 births (95% CI 503-1927) [3] but not as high as the Feusier et al. 2019 rate of 1:63 births (95% CI 30.6–153.8) (Fig. 2A). The large sample size in our study produces more reliable estimates with smaller confidence intervals than previous analyses (Fig. 2A), suggesting that our data provide the most accurate determination of TEI rates up to this time.

Recently published work on the same ASD cohort [12] detected fewer insertions and reported 31% (1 in 42 Alu), 49% (1 in 231 L1), and 33% (1 in 309 SVA) lower de novo insertion rates than ours, possibly due to their exclusion of mosaic insertions in their rate estimates, the use of a less sensitive pipeline [14], and not adjusting for the lower sensitivity for detection of TEIs in short-read data. We detected 47 visually inspected de novo candidates that were not reported by this previous analysis, and within the samples overlapping their cohort and the cohort of this study, we did not detect 27 of their de novo candidates. Only 13 of the 27 undetected events passed our criteria for visual inspection of having two breakpoints, a target site duplication, and a polyA tail while the rest either did not pass these criteria or had been excluded because they were considered inherited events. Most de novo candidates that we missed were not detected because they display few supporting reads, or the parental genomes have clipped reads near the breakpoint.

We demonstrated that the sensitivity and specificity of our pipeline are high (Additional file 1: Fig. S1) through rigorous benchmarking. Despite its comparable performance to MELT, we detected more novel TEIs compared to gnomAD-SV, which uses MELT to detect TEIs. It is likely because the post hoc filtering implemented in the gnomAD-SV study after running MELT [10, 18] reduces the sensitivity but increases the specificity in their analysis, resulting in fewer candidates compared to our analysis. Novel TEIs include candidates with both clipped and discordant read support for one breakpoint and either clipped or discordant read support for the other breakpoint. These “one-and-a-half” sided TEIs may not have a reported TSD but have sufficient read support to be considered high-confidence candidates by *xTea* (Additional file 1: Fig. S5A). This group may contain relatively more false positives than insertions with both clipped and discordant read support on both breakpoints as well as a TSD and a polyA tail, i.e., ‘two-sided tprt‘insertions. We have annotated and included both types of TEIs to maintain high sensitivity and so that we could provide a comprehensive dataset. All the reported candidates in this study have been uploaded to dbVar and users can customize based on the confidence classification rating (Additional files 4, 5, 6, 7, 8 and 9).

Assigning causality to non-coding variants based on clinical phenotypes is challenging, given that most known ASD genes have been discovered in the context of coding LoF variants, yet most individuals with ASD do not have LoF coding variants identified [9]. To understand the clinical phenotypes of individuals with TEIs in high pLI [36] or known ASD genes [21], we reviewed the available clinical data and compared this to any known phenotypes associated with the gene, as well as the scientific literature more generally available (Table 1). Exonic insertions are likely to disrupt the coding sequence and are thus of particular interest. We observed one exonic Alu insertion in *CSDE1*, which has been recently associated with ASD [22]. The affected proband shared clinical features, albeit non-specific, consistent with the previously described cohort, including ASD, intellectual disability, macrocephaly, and vision impairment. We additionally observed an exonic Alu insertion in *KBTBD6* (Table 1). Variation in this gene has not yet been associated with a reported neurodevelopmental phenotype that we are aware of. However, *KBTBD6* represents an intriguing candidate gene given its high pLI score (pLI = 0.935) [36] as well as its molecular interactions with known ASD gene *CUL3*, to mediate the activity of another ASD gene, *RAC1* [42]. Studying target genes of exonic de novo TEIs may shed novel biological insight not captured solely with more commonly studied forms of genetic variation in ASD.

We estimated a rate of underlying exonic TEIs of at least 1 in 2288 in ASD, which is similar to the rate of 1 in 2434 cases with developmental disorders reported in a recent exome sequencing study [5]. Although this is lower than other types of de novo genetic drivers of ASD, such as copy number variation, and the contribution of non-coding variants is thought to be smaller than coding LoF variants [10], the strong depletion of polymorphic TEIs in regulatory non-coding regions and enrichment of large de novo L1 insertions (~ 6 kb when full-length) in introns of ASD genes in cases but not in control suggest some of these non-coding events may contribute to ASD risk. Since intronic TEIs can affect gene function through various mechanisms, such as altering RNA expression and splicing [1], TEIs contributing to ASD may present a phenotype different from known phenotypes caused by LoF coding variants or large CNVs in these genes. Including TEIs and structural variants in standard clinical genetic analyses for ASD will continue to expand our knowledge of non-coding variants and could increase the rates of genetic diagnoses.

## Conclusions

We have established *xTea*, a scalable and sensitive method for detecting TEIs in WGS data on the Amazon cloud platform and applied it to a large cohort of > 8700 individuals from 2288 ASD quad families. Our work presents important advances in scalable bioinformatic processing of human WGS data and identification of TEIs, which by their nature represent a challenging form of genomic variation to study. We created a catalog of 86,154 polymorphic TEIs, a significant fraction of which were not previously reported. We shared the detailed features of each TEI as a community resource to enhance the understanding of TE biology and population genetics, and to facilitate the identification of disease associated TEIs. By leveraging the large cohort and rigorous pipeline benchmarking, we reported 158 de novo TEIs with robust rate estimates for each TE family. We discovered that although de novo rates between cases and controls are similar, cases had more de novo L1 TEIs in known ASD genes than expected. Most of these TEIs occurred in non-coding genic regions, suggesting that non-coding insertions could have a phenotypic impact. We also detected exonic TEIs in LoF genes in cases, including a causal exonic Alu insertion in *CSDE1*, known ASD gene [22]. Overall, our analysis suggests a modest, but important, impact of intronic and exonic TE insertions in ASD and implies the existence of diverse TEI-mediated pathogenic mechanisms beyond the insertional mutagenesis of protein-coding sequences. Future work, including both further development of computational methods, as well as experimental functional assessment of the effects and pathogenicity of non-coding TEIs, will be critical in understanding the role of these variants in ASD.

## Methods

### Datasets and data processing with *xTea*

Whole-genome data from the SSC from phases: Pilot, Phase 1, Phase 2, Phase 3-1, Phase 3-1, and Phase 4 were analyzed. The analyzed data consists of ASD families with one affected individual, two unaffected parents, and for 1860 of these families, one unaffected sibling was analyzed as the unaffected control. To process this massive amount of > 9000 individual whole genomes, we optimized for scalability a TEI detection computational tool, *xTea* [13] (https://github.com/parklab/xTea) and implemented a dockerized version on Amazon Web Services (Additional file 1: Table S8). After removing outlier results and confirming that these were due to corrupted bam files with incomplete sequences or failed *xTea* runs, we analyzed WGS data from ~ 2288 ASD affected individuals and ~ 1856 unaffected siblings with both parents sequenced (Additional file 1: Table S1 and Table S3 for sample sizes per TE type). The approximate average sequencing depth, as determined by *xTea*, was 39.4x. Paired-end reads were 151 base pairs in length.

### TEI identification with *xTea*

For each cram file, *xTea* ran three major steps to call TE insertions. First, raw candidate sites were collected based on whether there were enough qualified clipped reads at the breakpoints, where part of the read is aligned to the flanking region while the clipped part is well aligned to the consensus TE sequence. Second, for each passed candidate site we checked whether there was enough discordant reads support. Here, we consider a pair of reads with one read aligned to the flanking region and its mate aligned to the TE consensus sequence or other copies as discordant. Third, we ran TE-type specific filters to reduce false positives in both polymorphic and de novo insertions (see Additional file 1). *xTea* candidates were classified as “high” or “low” confidence insertions depending on whether enough insertion supporting features were distributed on both sides of the breakpoint. We only included insertions classified as “high confidence”*.*

### Annotation of non-redundant polymorphic TEIs

After obtaining the *xTea* high confidence insertions for each individual, we excluded calls where the clipped and discordant reads mapped above the consensus size *xTea* uses for mapping for AluY, L1HS, and SVA (282, 6120, and 1400 base pairs respectively). This removed some Alu insertions, which tended to be polyA expansion artifacts. Since breakpoint positions can have slight differences between individuals, these insertions were given a 40 base pair margin from the midpoint of the breakpoints and were merged if they overlapped to obtain a unique set of non-redundant TEIs in the SSC cohort.

To determine whether insertions in the SSC cohort were known or novel, merged TEI calls were overlapped with the breakpoints from gnomAD [18], 1000 genomes [14], or a compilation of other studies obtained from Evrony et al. [16] to obtain insertions in our cohort that are not found in these studies (novel), known TEIs which overlap, as well as known TEIs which overlap to individual studies only.

TSD sizes were obtained from the *xTea* output. The median size was selected for the merged insertions obtained with a 40 base-pair margin as described above. De novo candidates were analyzed separately. We also analyzed high-confidence TEIs classified by *xTea* as “two-sided target-primed reverse transcription (TPRT)”. These candidates are resolved on both breakpoints and have reads supporting a TSD and a polyA tail.

### Evaluating the performance of *xTea*

We created a haplotype-resolved dataset of non-reference TEIs in the Genome in a Bottle sample NA24385/HG002 [43] that has been sequenced with both long and short-read technologies. We evaluated the performance of *xTea* [13], MELT [14], and Mobster [15] using this dataset as the benchmark as described previously [13]. Briefly, we tested the sensitivity and specificity of these tools for detecting 1642 (1355 Alu, 197 L1, and 90 SVA) high-confidence TEIs detected in the NA24385/HG002 genome but not in the reference genome in pair-end downsampled Illumina WGS data. Note that the xTea version used in this study is different from the latest version reported in the xTea method paper [13].

### Calculation of TEI population allele frequency

The merged insertions were genotyped with the *xTea* genotyping module which uses a random forest model to genotype TEIs. We trained this model using 14 features from high confidence TEIs obtained from a subset of 1800 unaffected trio families from this cohort, as described previously [13]. If an insertion was detected in an unaffected child and only in one parent, and the other parent did not have any supporting clipped or discordant reads, it was labeled as heterozygous (0/1); if an insertion detected in a child did not have any supporting reads in the parental genomes, it was considered a false positive and labeled as reference homozygous (0/0); if an insertion was detected in both parents, had a ratio of discordant / (discordant + concordant) > 0.85, and no fully mapped reads at the breakpoint, it was labeled as homozygous (1/1). 70% of the insertion sites were used for training, and the accuracy in the remaining 30% of the sites was 99.7%. The population allele frequency (PAF) was calculated using only parental genomes in the cohort, which were unaffected and unrelated. Specifically, PAF for each polymorphic TEI was defined as the number of alleles carrying the TEI in the parents divided by the total number of chromosomes in the population (i.e.*,* 2 × the number of parents).

### Identification and rate estimation of de novo TEIs

To detect de novo insertions, we selected calls that did not have supporting reads in parental raw *xTea* output files and were confirmed via manual inspection. We further excluded de novo candidates which overlapped with reference and KNR TEIs (see Additional file 1). De novo retrotransposition rates were calculated as the number of de novo TEIs for both ASD affected and unaffected siblings divided by the total sample size. Samples that failed the *xTea* run were excluded from the analysis, resulting in a sample size of *n* = 4142 for L1, *n* = 4143 for Alu, and *n* = 4148 for SVA (Additional file 1: Table S3). Rates and confidence intervals from previous studies were obtained from Feusier et al. [4]. The 95% confidence intervals for de novo rates in the SSC cohort were obtained in the same manner, with an exact binomial confidence interval estimate.

We adjusted the observed de novo rates to account for sensitivity loss in short-read sequencing data and to obtain precise estimates. Specifically, we measured *xTea* sensitivity on the downsampled (39.4x) Illumina genome sequencing data from HG002, the HapMap sample extensively profiled by multiple sequencing platforms by the Genome in a Bottle consortium [43] using a high-quality catalog of haplotype-resolved non-reference TEIs for the sample (see Additional file 1 and Additional file 1: Fig. S1). We adjusted the number of total ASD and control de novo insertions by dividing the observed rate by the sensitivity for heterozygous TEIs, after filtering young reference and KNR TEIs, and obtained an exact binomial 95% confidence interval.

### Enrichment analysis using simulated TEIs

We performed simulations to calculate the probability of the number of observed insertions in ASD genes, high pLI (pLI ≥ 0.9) genes, or in different genomic regions occurring by chance (see Additional file 1). Using the observed de novo TEIs candidates in ASD and unaffected siblings and the number of unique polymorphic insertions in parents, we simulated the same number of insertions of the same size in random regions of the genome. We also performed these simulations considering the L1 endonuclease cleavage site preference, as described previously [37, 38]. Here, used the code provided by Wildschutte et al. (https://github.com/KiddLab/random-sample-by-ppm) to simulate random insertions using a position probability matrix corresponding to the L1 cleavage sites from HeLa cells [38]. We excluded the same young reference TE regions and KNR regions we excluded when detecting de novo TEIs for de novo simulations and excluding young reference TE regions for polymorphic insertions for both simulations. We performed 10,000 simulations and determined the number of random insertions that overlapped a SFARI gene, high pLI gene, or region of interest per simulation. We determined if the observed value fell on the upper or lower end of the observed distribution, with a pseudo count of 1, to obtain a *p*-value. For example, the upper *p*-value is defined as (r + 1)/(n + 1), where r is the number of simulations greater or equal to the observed value and n is the number of simulations. This value was multiplied by 2 for an empirical two-sided *p-*value. 95% CIs were calculated by obtaining the 0.025 and 0.975 percentiles of the null distribution. The log_2_ FC was calculated as the log_2_(observed value/mean of the null distribution) and the 95% CIs are plotted as their log_2_ values. These *p*-values were corrected for multiple testing with the Benjamini & Yekutieli method, to account for dependency between tests.

### Gene list enrichment in genes with TEIs

We tested whether genes with TEIs in ASD or controls were enriched for genes overexpressed in tissues in the Human Gene Atlas list using Enrichr [44]. We also tested for enrichment of gene ontology terms in the subset of genes with TEIs using g:Profiler [45] in only annotated genes, with a user threshold of 0.05 and a significant threshold for multiple testing correction with the g:SCS threshold method.

### PCR validations

Twenty four cases were chosen for full-length PCR validation based on their clinical relevance by selecting variants that occurred in SFARI, high pLI, or brain expressed genes and 13 cases were randomly selected for a total of 12 L1 insertions and 25 Alu insertions (Additional file 3: Table S7). We developed a pipeline for designing specific primers and tested and optimized the PCR protocols for each primer pair in control DNA before validating them in the SSC samples (Additional file 1). Out of 12 L1 primer pairs designed for validations of de novo insertions, we were able to optimize 9 primer pairs, and we optimized 23 Alu primer pairs out of 25. Two of the L1 primers were selected for mosaic candidates in 1 case and 1 control and were considered separately for validation rates. Validations were performed with 20 ng of DNA from each available family member from lymphoblastoid cell lines which were provided by the Rutgers University Cell and DNA Repository. This was done by confirming the presence of both an insertion and a non-insertion allele band near or at the expected insertion size in the samples with predicted insertions, and only a non-insertion allele band in the other family members. Water was used instead of DNA as a non-template control for each primer pair.

## Supplementary Information

**Additional file 1: Supplementary Methods**, **Figure S1.** The version of *xTea* used in this study has a high performance and is comparable to MELT in short-read Illumina WGS data. **Figure S2.** Polymorphic and de novo transposable element insertions (TEIs) in the SSC cohort. **Figure S3.** The number of Alu TEIs detected per individual is different for some populations. **Figure S4.** Percentage of insertions which were not found in previous studies (novel) or overlap with TEIs from previous analyses (known). **Figure S5.** Target site duplication (TSD) size distribution for de novo and polymorphic novel and known non-reference (KNR) TEIs. **Figure S6.** Comparison of population allele frequencies (PAFs) between unrelated parental individuals in the SSC cohort and gnomAD-SV TEIs. **Figure S7.** The observed number of de novo TEIs in high probability of being loss of function intolerant (pLI) genes compared to expected TEIs based on 10,000 random simulations. **Figure S8**. The observed number of de novo TEIs in SFARI ASD genes and high pLI genes compared to expected TEIs based on 10,000 random simulations using a position probability matrix to consider the L1 endonuclease cleavage site. **Figure S9**. Parental age at birth of children with and without TEIs for cases and controls combined. **Figure S10.** Estimated insertion size of TEIs. **Figure S11.** Enrichment and depletion of TEIs in coding and gene regulatory regions. **Figure S12.** Enrichment and depletion of TEIs in coding and gene regulatory regions. **Table S1.** Polymorphic insertions sample sizes. **Table S3.** De novo insertion rates and sample sizes. **Table S4.** De novo insertions that overlap the top 10% expressed genes in the neocortex during development. **Table S5.** Number of de novo insertions overlapping regions with epigenetic annotation in fetal brain. **Table S6.** Number of observed polymorphic insertions in parental SSC samples overlapping regions with epigenetic annotation in fetal brain. **Table S8.** Memory and time cost of xTea on different numbers of CPU cores.

**Additional file 2: Table S2**: De novo insertions in ASD and controls.

**Additional file 3: Table S7**: PCR validations primers and samples.

**Additional file 4.** Alu polymorphic and de novo TEIs for the entire cohort intervals after merging with a 40 base-pair margin.

**Additional file 5.** L1 polymorphic and de novo TEIs for the entire cohort intervals after merging with a 40 base-pair margin.

**Additional file 6.** SVA polymorphic and de novo TEIs for the entire cohort intervals after merging with a 40 base-pair margin.

**Additional file 7.** Alu polymorphic and de novo TEIs for unrelated parents in the cohort after merging with a 40 base-pair margin.

**Additional file 8.** L1 polymorphic and de novo TEIs for unrelated parents in the cohort after merging with a 40 base-pair margin.

**Additional file 9.** SVA polymorphic and de novo TEIs for unrelated parents in the cohort after merging with a 40 base-pair margin.

## Data Availability

The parental high-confidence TEIs from our study are available in the dbVar repository under the study accession “nstd203”, https://www.ncbi.nlm.nih.gov/dbvar/?term=nstd203. A vcf of all TEIs identified with *xTea* and their annotations can be found here: https://ftp.ncbi.nlm.nih.gov/pub/dbVar/data/Homo_sapiens/by_study/genotype/nstd203. We also created two merged TEI sets from all the TEIs reported in the vcf file—one from all SSC individuals and the other only from parental genomes—for each TE family. The TEI sets along with TSD size, estimated insertion size, genotype, and confidence classifications are available as supplementary material (Additional files 4, 5, 6, 7, 8 and 9). A 40-bp margin was used to merge TEIs since the predicted breakpoints can slightly differ even for the same TEI depending on the supporting clipped reads in each sample, resulting in the 86,154 reported insertions. The most current version of *xTea*, including the filtering and genotyping module used in this study after running the initial pipeline, can be found here: https://github.com/parklab/xTea/. The docker file and version (warbler/xteab:v9) used in our analysis can be found here: https://hub.docker.com/repository/docker/warbler/xteab and the GitHub *xTea* branch used is located here: https://github.com/parklab/xTea/tree/release_xTea_cloud_1.0.0-beta. This software is platform-independent and the programming language is Python. The license and additional requirements can be found here: https://github.com/parklab/xTea/. The code used for detection of de novo insertions and for designing primers for full-length validation of TEIs is located here: https://github.com/ealeelab/TEs_ASD. The raw sequencing data that support the findings of this study are available from the Simons Foundation Autism Research Initiative, but restrictions apply to the availability of these data and so are not publicly available. Access may be requested through https://base.sfari.org/.
