# Supplementary material for: Whole-genome analysis reveals the contribution of non-coding de novo transposon insertions to autism spectrum disorder
Source: Mob DNA. 2021 Nov 27;12:28. doi: 10.1186/s13100-021-00256-w (PMC8627061; doi:10.1186/s13100-021-00256-w)
Supplement: Supplementary file 1 — Additional file 1: Supplementary Methods, Figure S1. The version of xTea used in this study has a high performance and is comparable to MELT in short-read Illumina WGS data. Figure S2. Polymorphic and de novo transposable element insertions (TEIs) in the SSC cohort. Figure S3. The number of Alu TEIs detected per individual is different for some populations. Figure S4. Percentage of insertions which were not found in previous studies (novel) or overlap with TEIs from previous analyses (known). Figure S5. Target site duplication (TSD) size distribution for de novo and polymorphic novel and known non-reference (KNR) TEIs. Figure S6. Comparison of population allele frequencies (PAFs) between unrelated parental individuals in the SSC cohort and gnomAD-SV TEIs. Figure S7. The observed number of de novo TEIs in high probability of being loss of function intolerant (pLI) genes compared to expected TEIs based on 10,000 random simulations. Figure S8. The observed number of de novo TEIs in SFARI ASD genes and high pLI genes compared to expected TEIs based on 10,000 random simulations using a position probability matrix to consider the L1 endonuclease cleavage site. Figure S9. Parental age at birth of children with and without TEIs for cases and controls combined. Figure S10. Estimated insertion size of TEIs. Figure S11. Enrichment and depletion of TEIs in coding and gene regulatory regions. Figure S12. Enrichment and depletion of TEIs in coding and gene regulatory regions. Table S1. Polymorphic insertions sample sizes. Table S3. De novo insertion rates and sample sizes. Table S4. De novo insertions that overlap the top 10% expressed genes in the neocortex during development. Table S5. Number of de novo insertions overlapping regions with epigenetic annotation in fetal brain. Table S6. Number of observed polymorphic insertions in parental SSC samples overlapping regions with epigenetic annotation in fetal brain. Table S8. Memory and time cost of xTea on different numbers of CPU [file 13100_2021_256_MOESM1_ESM.pdf]

Supplemental Material and Methods

Data processing on the cloud with *xTea*

The Simons Simplex Cohort (SSC) samples are hosted on Amazon Web Services (AWS) and Tibanna (1) was used for managing jobs on AWS. For each job, a cloud instance with specific configurations was created, and each cram file was downloaded from the S3 bucket to the instance. *xTea* docker was pulled from docker hub (https://hub.docker.com/repository/docker/warbler/xteab, v9). The reference genome and repeat libraries were also downloaded from the S3 bucket to each instance. *xTea* was run on each downloaded cram, and the results were compressed and saved to the Amazon S3 bucket. The running cost varies based on memory usage and the number of cores. We evaluated the performance of *xTea* with different numbers of cores and memory (Additional File 1: Table S8) and used a spot instance with 16 cores and 32G memory (c5.4x large) for all samples.

TE filtering with *xTea*

TE type-specific filters were implemented within *xTea* to remove false positives. In specific, while we used the default values for most of the parameters, there are three major parameters (the number of clipped reads, the number of discordant pairs, and the number of clip and discordant reads) which can affect the sensitivity and specificity. We thoroughly evaluated these three parameters and required >= 3 clipped reads, >=5 discordant pairs, and >=1 clip and discordant pairs as the optimal ones to maintain high sensitivity and high specificity. Because of target-primed reverse transcription, a polyA tail and target site duplication should be observed along with enough supporting clipped and discordant reads at both sides of the breakpoint. However, in many cases, not all of these features could be detected. *xTea* incorporates a confidence rating system that evaluates whether all these features are found and whether they are on one or both sides of the breakpoint. We selected only insertions classified as “high

confidence”. Additional filters within *xTea* include examining the patterns of insertion-supporting clipped sequences and discordant reads mapped to the TE consensus sequences: the supporting reads should not be scattered across the consensus but instead form one cluster (c1) for 5’-clip reads and another cluster (c2) for 3’-clip reads; the mates of 3’ and 5' discordant reads should form two distinct clusters (d1 and d2). The distance between c1 and d2 and between c2 and d1 must be less than the average insert size ± 3*(standard deviation of the insert size). The supplementary *xTea* filter module with TE type-specific filters implemented in our analysis is now part of the main *xTea* code in the latest version.

Processing for comparison with known non-reference TEIs

To obtain Venn diagrams for overlap with other cohorts, TEIs from unrelated parental individuals were given a 40 base pair margin from the midpoint of the breakpoints and were merged with bedtools (2) “merge” if they overlapped to obtain a unique set of non-redundant TEIs in unrelated individuals in the SSC cohort. Breakpoints from gnomAD (3) and 1000 genomes (4) were also given a 40 base pair margin. The different datasets were overlapped using bedtools (2) “intersect” and counts were plotted in R with the VennDiagram library (5).

Allele frequencies and comparison with previous studies

The population allele frequency (PAF) within the cohort was calculated as the number of alleles carrying the TEI in the population divided by the total number of chromosomes in the population. As an additional approach, we estimated the population allele frequency (PAF) within the SSC parental cohort using the Hardy-Weinberg principle. Here, p+q=1, where p is the frequency of the insertion allele in the population and q is the frequency of the non-insertion allele in the population. Assuming that q^2 is the fraction of individuals without an insertion, we calculated the PAF as 1 - (sqrt((total parental individuals in the cohort - individuals with insertion allele) / total individuals in the cohort)). Merged breakpoints were overlapped with gnomAD TEIs (3) using a

window of 40 base pairs to define overlap. We compared the PAF within the SSC cohort to both the overall PAF and the European PAF in gnomAD since 83% of fathers and 85% of mothers were classified as white (Additional File 1: Fig. S6).

Detection of *de novo* TEIs

We used the high confidence post-filtered insertions from *xTea* (6) for this analysis for Alu, L1, and SVA (https://github.com/parklab/xTea). TEIs were given a 40 base pair margin from the midpoint of the breakpoints and were excluded if they overlapped with known non-reference (KNR) insertions obtained from previous studies (4, 7-14) as well as reference SVA, reference young L1 (L1HS, L1PA2, L1PA3) or reference young Alu (AluY) (15). To exclude inherited insertions that may have been missed in parents, we excluded insertions that had clipped or discordant reads in the raw parental files (clip_reads_tmp0 and discordant_reads_tmp0) in the *xTea* output. We imaged *de novo* candidates on IGV 2.4.19 (16) for manual inspection. We visually confirmed the absence of supporting parental reads, as well as the presence of a target site duplication, a polyA tail, and clipped and discordant supporting reads that support a retrotransposition event (17). Insertions were scored as "high confidence *de novo*" if visual inspection of calls passed these criteria for *de novo* insertions, as "*de novo*" if there were some discordant reads in the parents but no clipped reads supporting the breakpoint in parents, as "somatic candidate" if there is strong read support with a polyA tail, target site duplication, clipped reads and discordant reads, but the supporting reads are a small fraction of the overall coverage at the breakpoint, as "parental mosaic candidate" if there were <=2 clipped reads in one of the parents and discordant reads at a low allele frequency, suggesting it might be mosaic in parent's blood yet not called by *xTea* due to the low allele frequency, and "false negative parental" if it was not clear whether there was a false negative insertion or a mosaic blood insertion in the parental sample due to having few clipped reads but many discordant reads near the insertion site. Only insertions scored as "high confidence *de novo*", "*de novo*", "somatic

candidate", and "parental mosaic candidate" were included. TEIs that were detected in both the affected and unaffected siblings were excluded as these are either parental false-negative insertions or parental mosaic and inherited events.

Adjusting *de novo* retrotransposition rates

With long-read technologies, the sensitivity for detection of TEIs is higher (18, 19), suggesting that our raw rates are an underestimate. To account for genomic regions in which *xTea* is unable to detect TEIs given the lower sensitivity with Illumina short-read data, as well as for the reference filters we used for *de novo* insertions, we calculated our sensitivity for detecting germline TEIs in the Genome in a Bottle sample NA24385/HG002 (20) which has been sequenced with both long and short-read technologies. A curated set of 9,970 (>50bp) insertions was obtained from Genome in a Bottle V0.6 (21) and integrated with 15,268 (>50bp) insertions from a haplotype assembly of the samples (6, 22). RepeatMasker (15) was used to annotate Alu, L1, and SVA sequences, and then insertions were confirmed by manual inspection of poly-A tails and target site duplication or deletions on IGV (23). This resulted in 1,642 (1,355 Alu, 197 L1, and 90 SVA) high-confidence TEIs detected in the NA24385/HG002 genome but not in the reference genome. We downsampled the HG002 Illumina bam file to the average coverage of SSC samples (39.4x) and detected TEIs with *xTea*. We excluded calls that overlapped reference SVA, reference young L1 (L1HS, L1PA2, L1PA3), or reference young Alu (AluY) (15), as performed for the SSC analysis and calculated the sensitivity of our pipeline to detect the set of curated TEIs.

Annotation of TEIs

UCSC Table Browser (24) was used to obtain RefSeq gene annotations and coordinates for exons, and introns. ASD gene annotations were obtained from Simons Foundation Autism Research Initiative (SFARI) gene (25) in March 2019. Categories S (Syndromic),1 (High

Confidence), 2 (Strong candidate), 3 (Suggestive evidence), 4 (Minimal evidence), and 5 (Hypothesized but untested) were included. *De novo* insertion candidates were also annotated with the probability of being loss-of-function intolerant (pLI) (26).

Chromatin states from fetal brain tissue (E081 and E082) were downloaded from https://egg2.wustl.edu/roadmap/data/byFileType/chromhmmSegmentations/ChmmModels/imputed12marks/jointModel/final/ (27). States were classified as: 13_EnhA1 and 12_EnhA2 = active enhancers; 15_EnhAF, 16_EnhW1, 17_EnhW2, 18_EnhAc = other enhancers (weak, flank, acetylation only); 2_PromU, 3_PromD1, 4_PromD2 = promoters. TEIs were overlapped with the two fetal brain regions using bedtools (2) and the number of unique calls in each category was obtained.

Mobile Element Insertion Size

To determine the size of polymorphic and *de novo* insertions, we only included TEIs which had supporting clipped reads on both breakpoints and which did not overlap with reference. Since *xTea* maps reads to several subfamilies of retrotransposons, we excluded calls where the clipped and discordant reads mapped above the consensus size *xTea* uses for mapping for AluY, L1HS, and SVA (282, 6,120, and 1,400 base pairs respectively) since, particularly for Alu calls, these tended to be poly-A expansion artifacts. The consensus sequences within *xTea* were obtained from RepBase23.02 (28) and the L1HS consensus sequence was manually constructed by multiple sequence alignment of full-length reference sequences. The position of clipped and discordant reads mapping to reference retrotransposon sequences was obtained for each insertion. The minimum position was subtracted from the maximum position to obtain the predicted size. If the maximum position was larger than the consensus length, this was set to the consensus length. The resulting estimated insertion size is an approximation since we are unable to account for repeat expansions and different poly-A tail lengths (29, 30). For all insertions including polymorphic and *de novo* TEIs, calls were given a 40 base pair margin from

the midpoint of the two insertion breakpoints and were merged based on the overlap of these coordinates. The median size for all samples with each insertion is reported. Loess regressions were performed in R (31) with a 25% smoothing span.

De novo insertions in brain expressed genes

For overlap of insertions with brain expressed genes, we selected the neocortex regions from Brainspan (32, 33) (ventrolateral prefrontal cortex (VFC), dorsolateral prefrontal cortex (DFC), medial prefrontal cortex (MFC), primary visual cortex (V1C), primary motor cortex (M1C), orbitofrontal cortex (OFC), primary association cortex (A1C), inferior parietal cortex (IPC), primary somatosensory cortex (S1C), superior temporal cortex (STC), inferior temporal cortex (ITC)) (34), and obtained the mean expression of each gene per sample in these tissues and then obtained the mean expression per age group for the following categories: Early prenatal: 8-19 PCW, Late prenatal: 21-37 PCW, Childhood (4 months -11 years), Adolescence: 13-19 years, and Adulthood: 21-40 years (Additional File 1: Table S4). We then overlapped genes with insertions in Autism Spectrum Disorder (ASD) and controls with the top 10% of gene expression observed.

Primer design and validation strategy

We excluded events overlapping duplicated regions or reference insertions of the same class from validations to reduce amplification artifacts. A custom pipeline, based on a previously developed pipeline (35), was used to obtain primer sequences for full-length validation. Sequences from -800 to -100 and +100 to +800 base pairs from the insertion breakpoint were used to select primers with Primer3 (36). InSilico PCR from UCSC (https://genome.ucsc.edu/cgi-bin/hgPcr) was then implemented to assess whether these primers would amplify a unique region in the genome. Blat (37) (-stepSize=5 -minScore=20 -minIdentity=80) was then used to confirm unique mapping to the genome. If these steps failed,

a masked genome (https://hgdownload.cse.ucsc.edu/goldenpath/hg38/bigZips/hg38.fa.masked.gz) was used for the first step.

PCRs were performed using Phusion Hot Start II High-Fidelity DNA Polymerase (F549L, Thermo Fisher Scientific) (see Additional File 3: Table S7 for detailed PCR protocols). Primers were tested and optimized using the Genome in a Bottle sample NA24385/HG002 (20), where we had sequencing data and high confidence insertions from the gold standard available. DNA was quantified using a Quant-iT™ dsDNA Assay Kit (Q33120, Thermo Fisher Scientific) before running at least 70 ng of PCR product, when possible, on a 2% agarose gel (for Alu) or a 1% agarose gel and with a Genomic DNA ScreenTape Analysis (5067-5366, Agilent) on an Agilent TapeStation (for L1) for a higher resolution at determining the insertion amplicon size. A 1kb Plus DNA ladder (10787-026, Invitrogen) was used.

Some primer pairs produced additional amplification bands or artifact bands and were further optimized by increasing the annealing temperature and/or decreasing the number of amplification cycles, and some primer pairs produced lower concentrations of DNA and were optimized by increasing the number of amplification cycles and/or decreasing the annealing temperature. If primer pairs did not amplify a unique non-insertion allele and had artifact bands, we did not proceed with validation of those insertions using those primers. Out of 12 L1 primer pairs designed for validations of *de novo* insertions, we were able to optimize 9 primer pairs, and we optimized 23 Alu primer pairs out of 25. 2 of the L1 primers were selected for mosaic candidates in 1 case and 1 control and were considered separately for validation rates. These 2 cases did not validate in lymphoblastoid cell line DNA.

Supplemental Figures

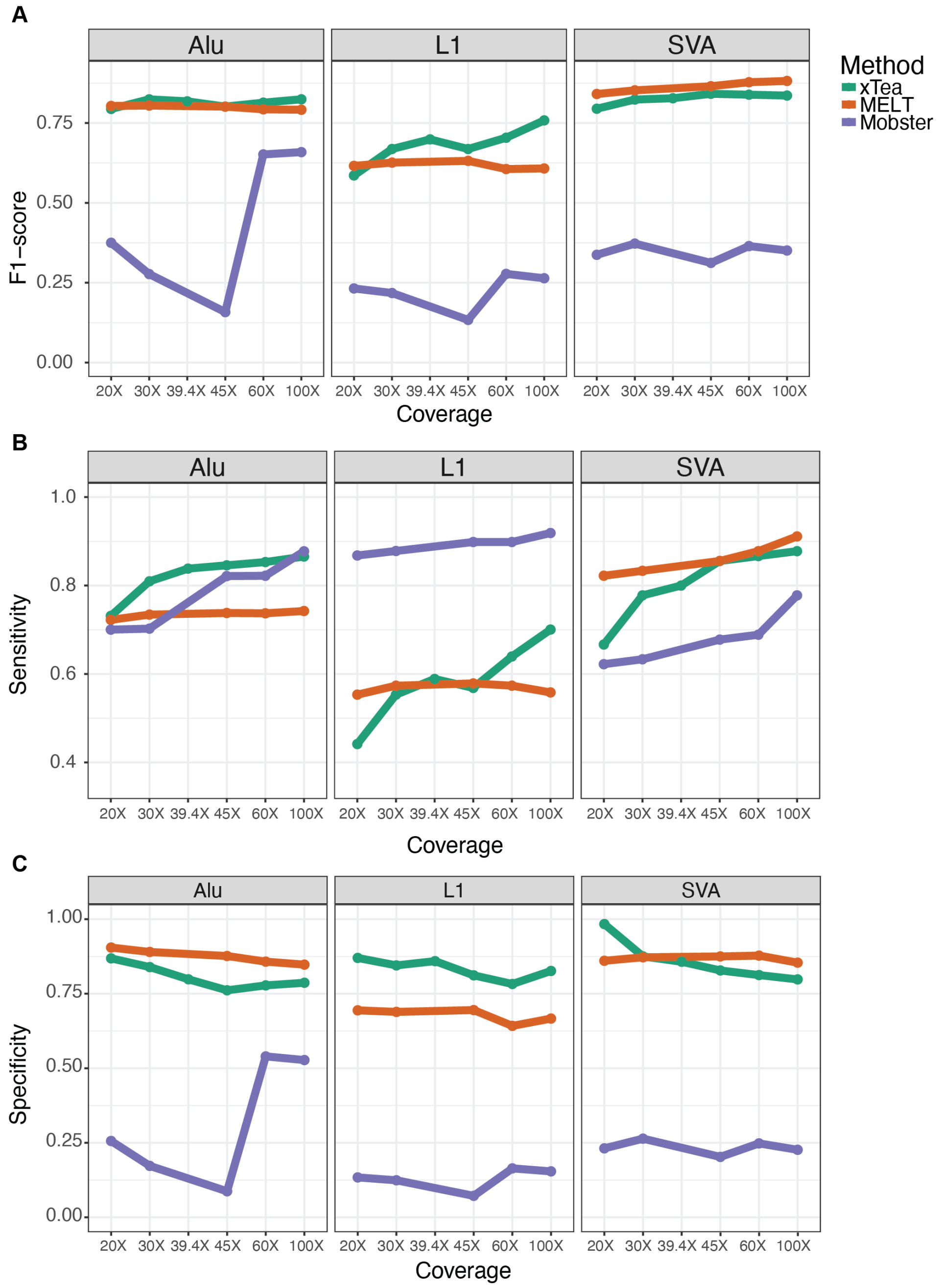

Fig. S1. The version of *xTea* used in this study has a high performance and is comparable to MELT in short-read Illumina WGS data. **A** *xTea* and MELT show higher F1 scores for Alu, L1, and SVA TEIs compared to Mobster. **B** At 40X coverage, the sensitivity of *xTea* is higher for Alu and is comparable to MELT for SVA and L1. The sensitivity of Mobster is higher for L1, at the cost of lower specificity. **C** The specificity of MELT and *xTea* is comparable for Alu and SVA at 40X and is higher in *xTea* for L1. Mobster shows a much lower specificity for Alu, L1, and SVA.

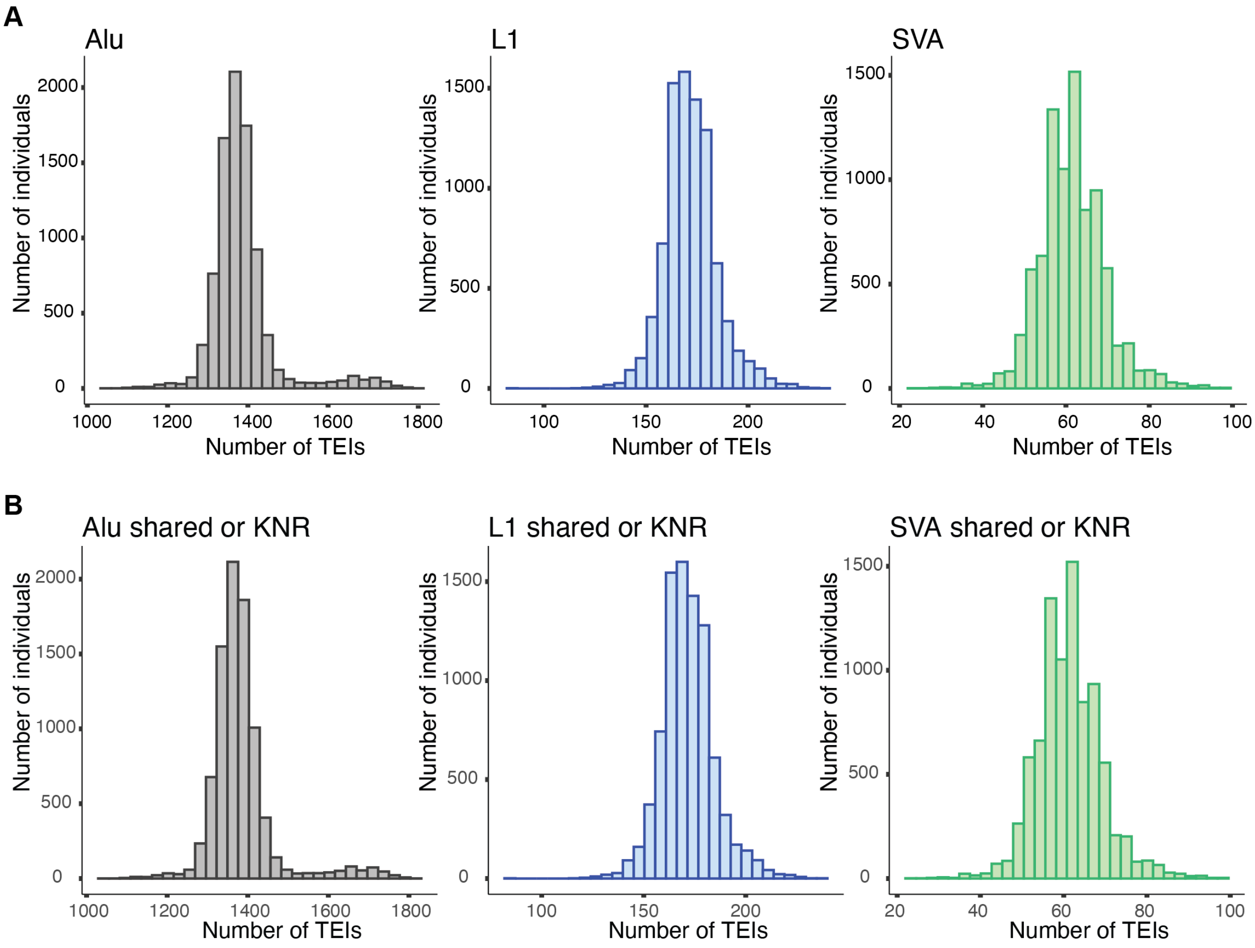

Fig. S2. Polymorphic and *de novo* transposable element insertions (TEIs) in the SSC cohort. **A** Number of TEIs detected per individual including parental, ASD, and unaffected siblings. Alu N=8,711, mean=1385.45, SD=82.12; L1 N=8,714, mean=171.94, SD=13; SVA N=8,720, mean=61.50, SD=7.75. **B** Shared polymorphic and known non-reference TEIs in the SSC cohort. Number of TEIs detected per individual including parental, ASD, and unaffected siblings which are found in more than 2 individuals and/or in gnomAD (3) or 1000 genomes (4). Alu N=8,711, mean=1383.26, SD= 81.23; L1 N=8,714, mean=171.62, SD= 12.89; SVA N=8,720, mean=61.36, SD=7.72.

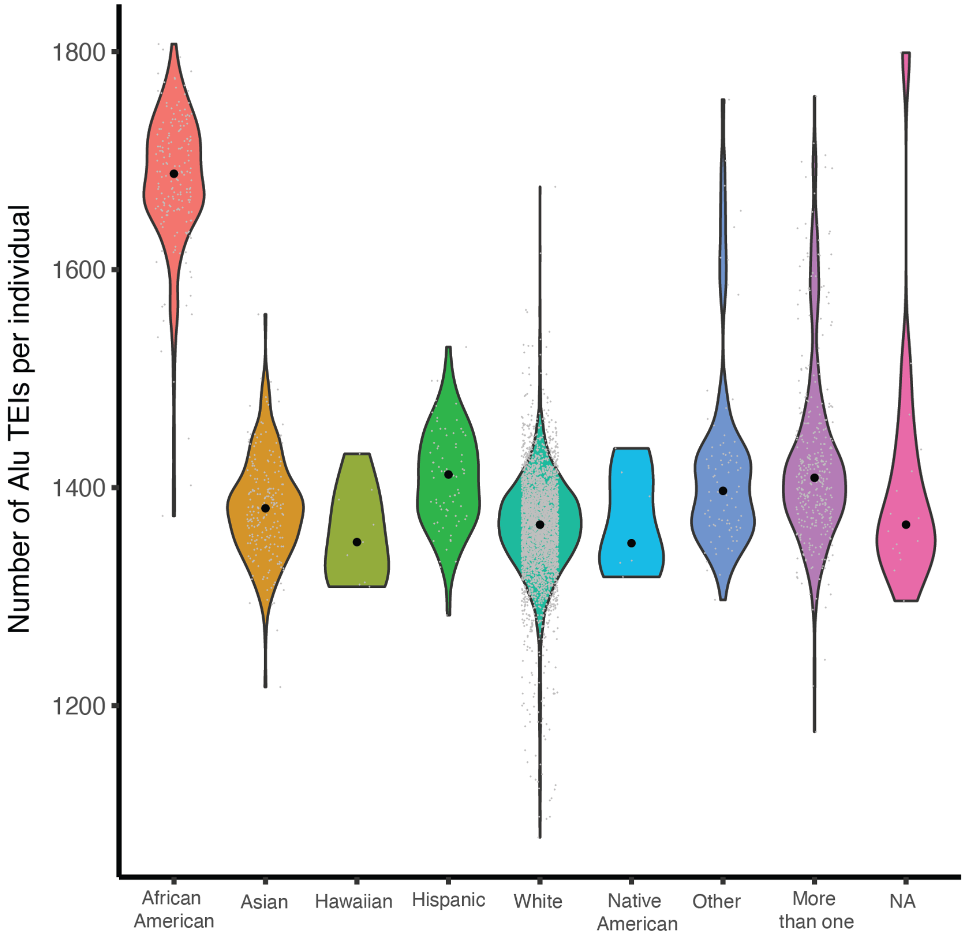

Fig. S3. The number of Alu TEIs detected per individual is different for some populations. Each grey dot represents the number of polymorphic and *de novo* detected per individual. The black dot is the median.

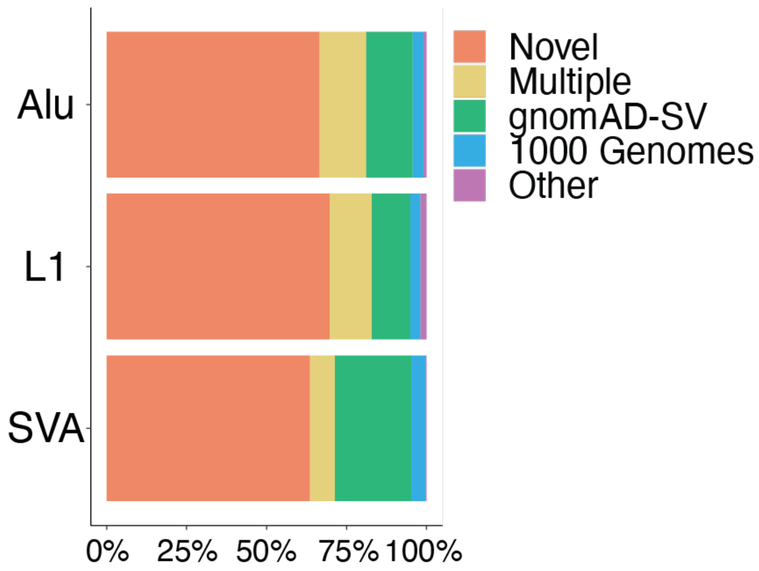

Fig. S4. Percentage of insertions which were not found in previous studies (novel) or overlap with TEIs from previous analyses (known) subdivided by the source of known overlap (3, 4, 7-14).

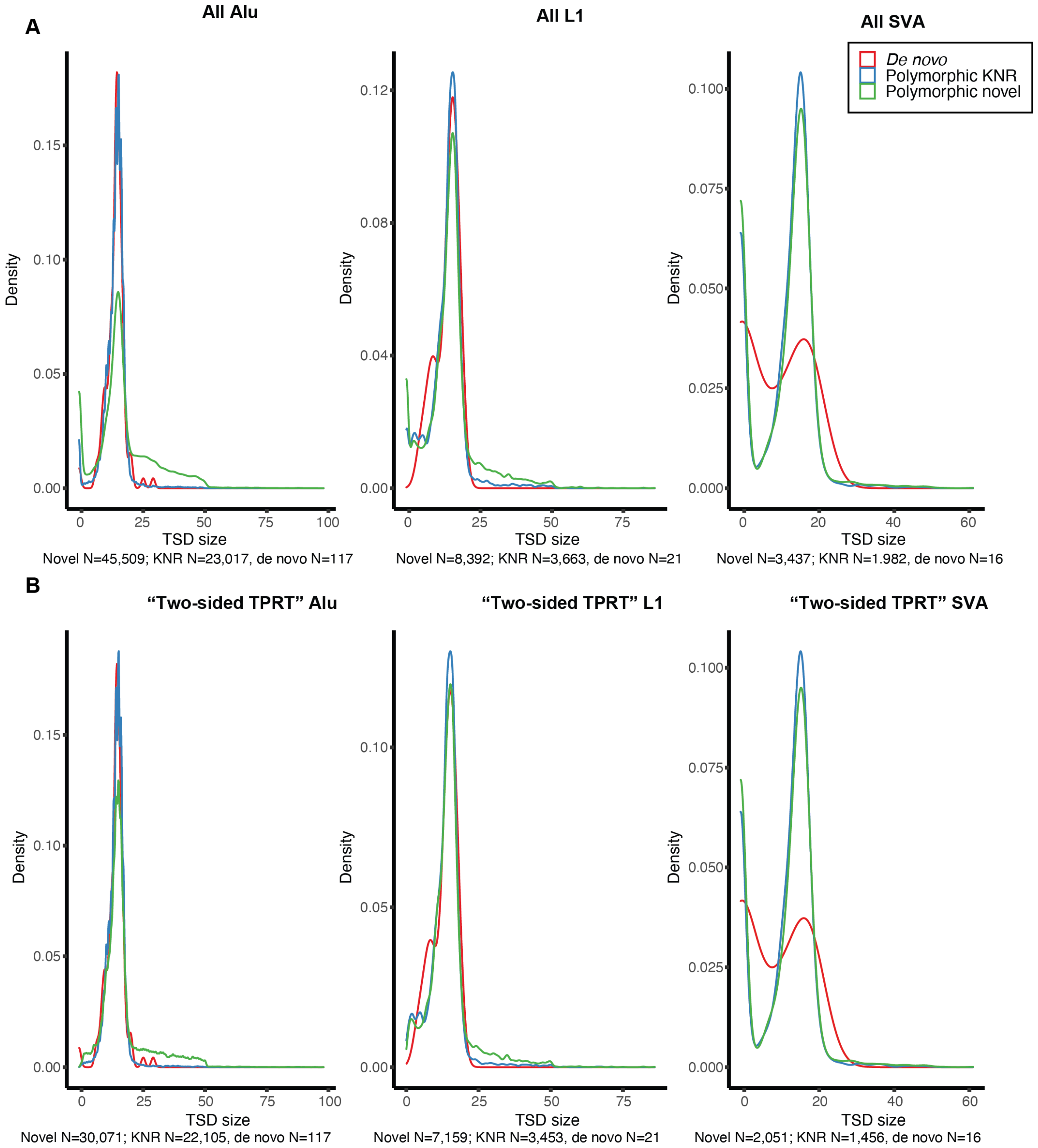

Fig. S5. Target site duplication (TSD) size distribution for *de novo* and polymorphic novel and known non-reference (KNR) TEIs. **A** L1 and SVA novel TEIs have a similar distribution to KNR insertions. Novel Alu TEIs contain more candidates without a detected TSD (-1 size) or with a larger TSD. **B** Novel insertions in the highest confidence category with both breakpoints resolved, a TSD, and a polyA tail (called two-sided Target-Primed Reverse Transcription (TPRT) in *xTea*), show a similar TSD size distribution to KNR insertions.

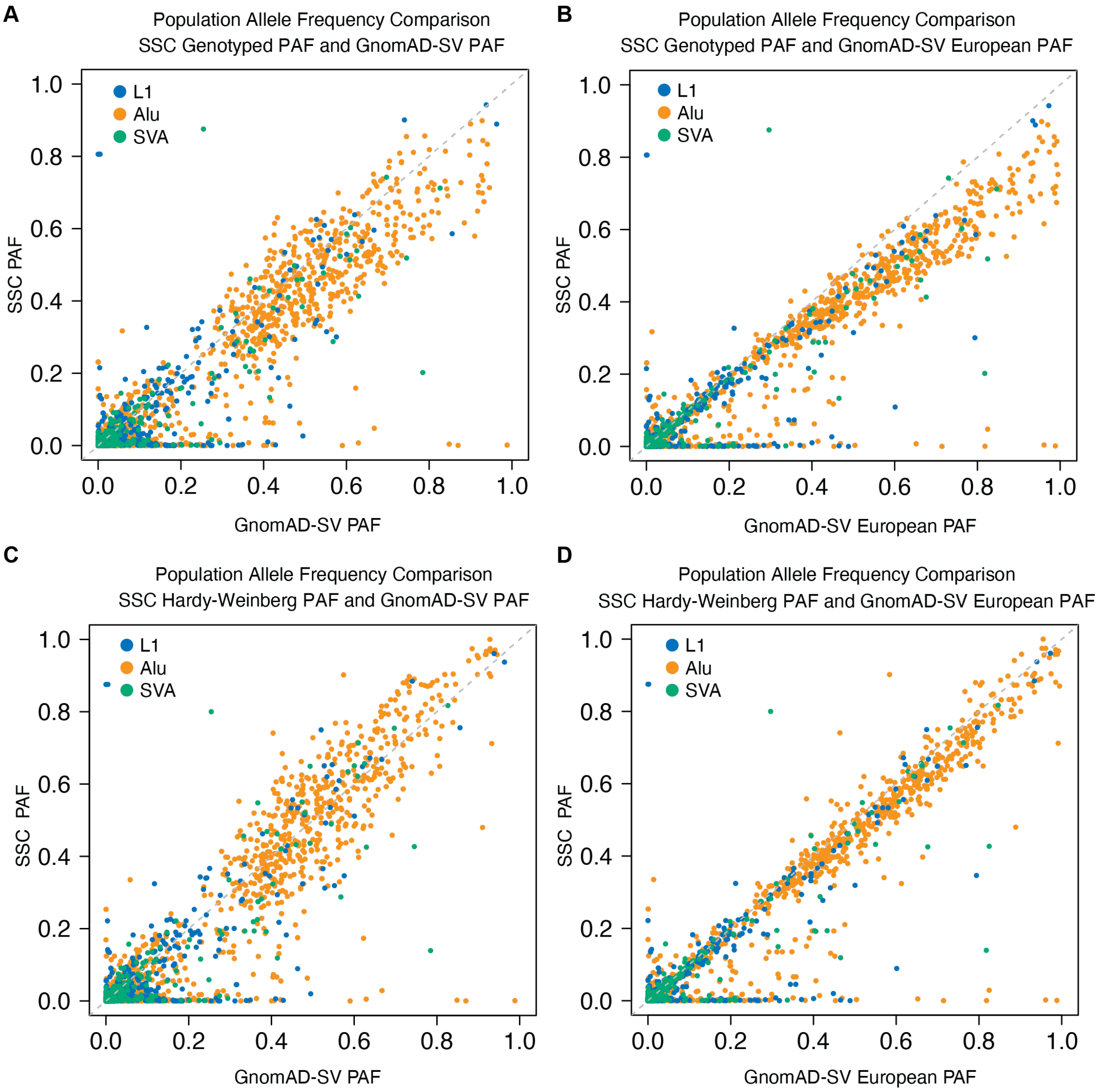

Fig. S6. Comparison of population allele frequencies (PAFs) between unrelated parental individuals in the SSC cohort and gnomAD-SV TEIs (3). **A** Comparison using the SSC PAFs from genotyped TEIs. The PAF was defined as the number of alleles carrying the TEI in the population divided by the total number of chromosomes in the population. GnomAD-SV PAFs are for the entire population or **B** the European population. **C** Same comparison as in Fig. S6A with SSC parental PAFs estimated using the Hardy-Weinberg principle compared to gnomAD-SV PAFs in the entire population and **D** gnomAD-SV European PAFs.

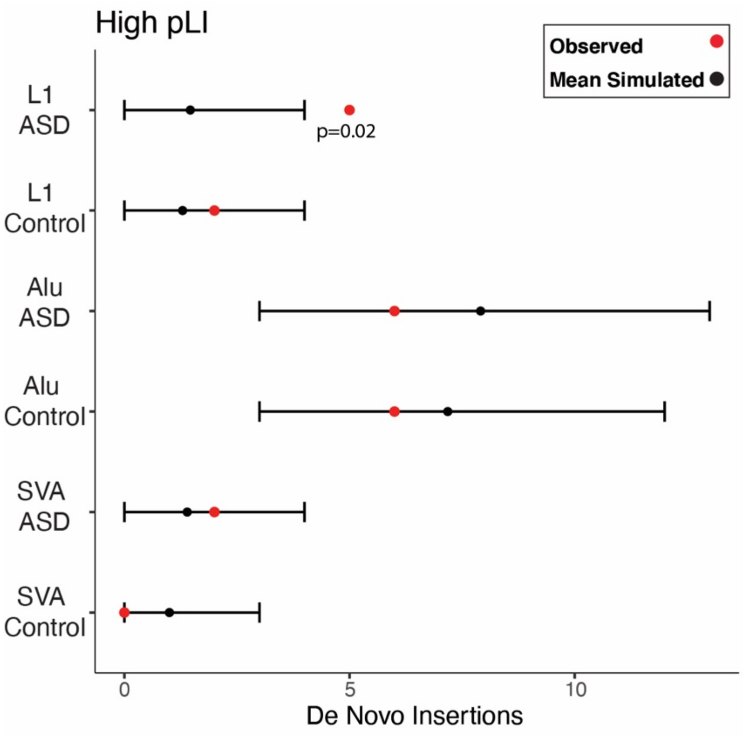

Fig. S7. The observed number of *de novo* TEIs in high probability of being loss of function intolerant (pLI) genes (26) (pLI >=0.90) compared to expected TEIs based on 10,000 random simulations. A trend for L1 insertions in high pLI genes than expected is observed in cases (not significant after multiple testing correction with the Benjamini & Yekutieli method).

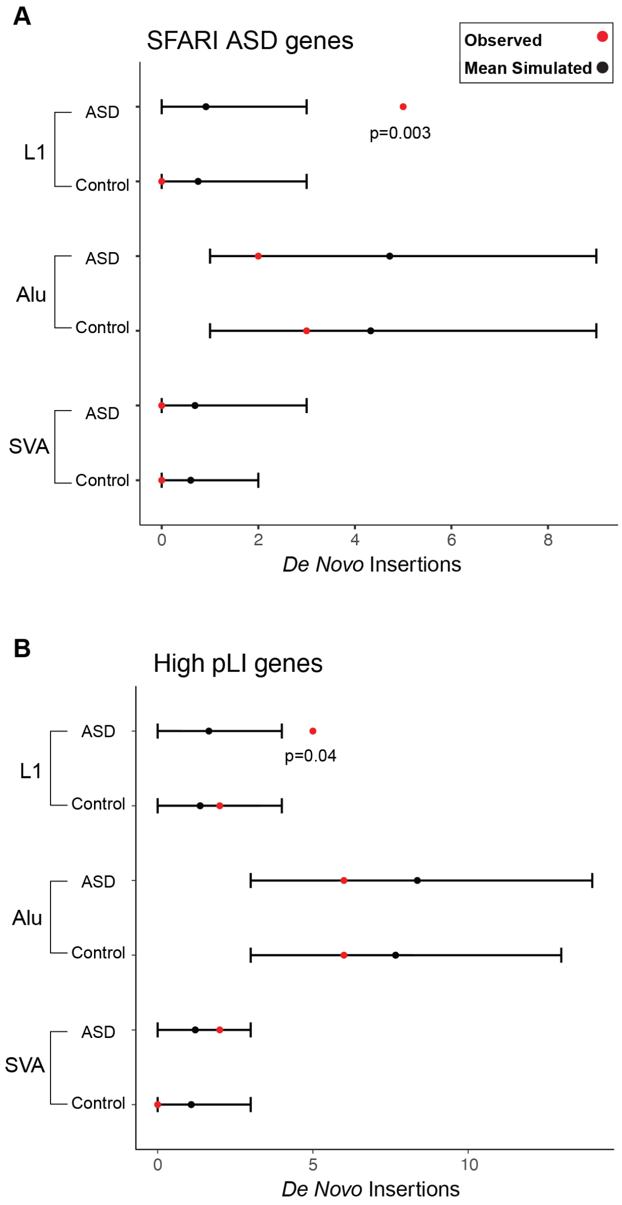

Fig. S8. The observed number of *de novo* TEIs in SFARI ASD genes and high pLI genes (26) (pLI >=0.90) compared to expected TEIs based on 10,000 random simulations using a position probability matrix to consider the L1 endonuclease cleavage site. **A** Similar results to the simulations performed with fully random insertions (Fig. 3) were observed, including a trend for more L1 insertions in SFARI genes in cases than expected by chance (p=0.003, q-value = 0.07). **B** We also identified a non-significant trend for more high pLI genes than expected in cases with this method (p=0.04, q-value = 0.52).

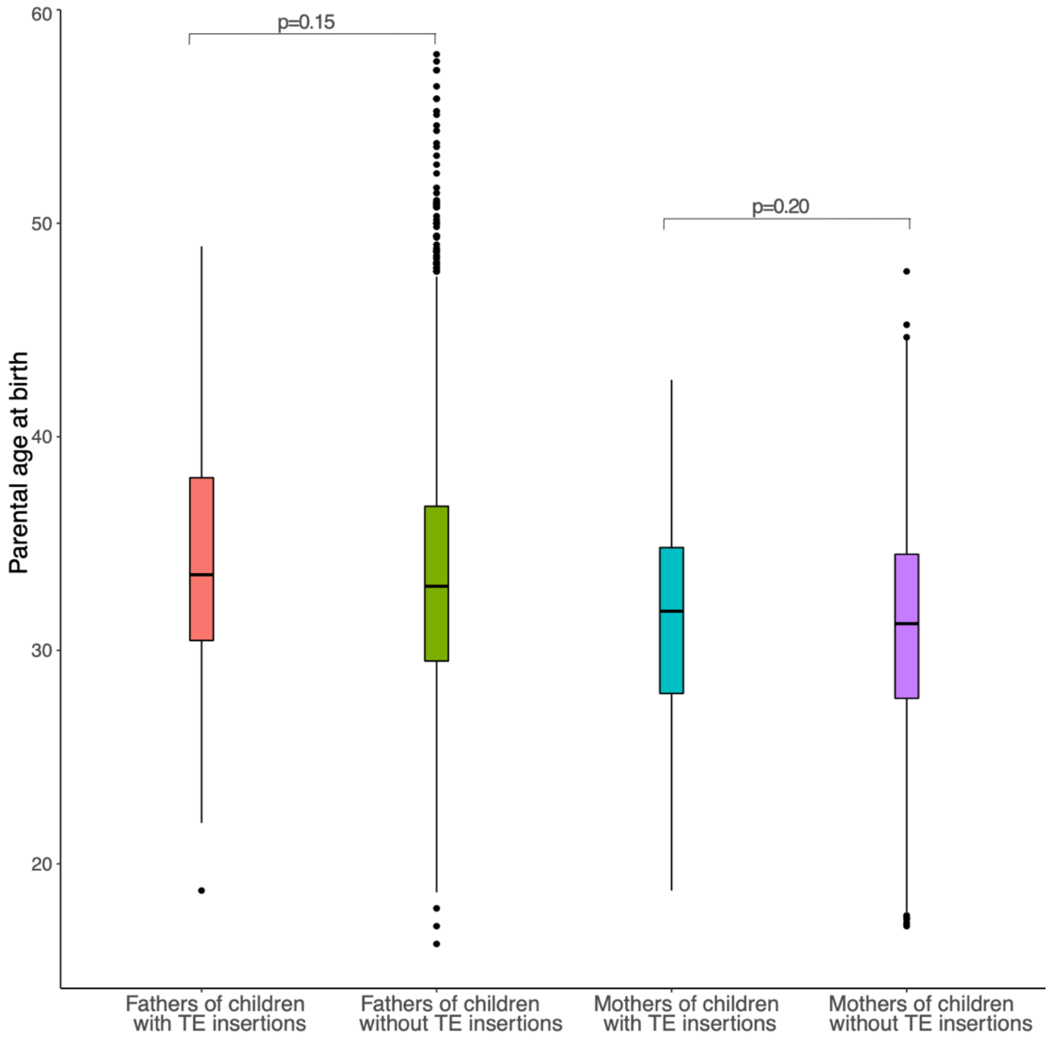

Fig. S9. Parental age at birth of children with and without TEIs for cases and controls combined. The median is represented with a line in the middle of the box plot and dots represent outlier samples.

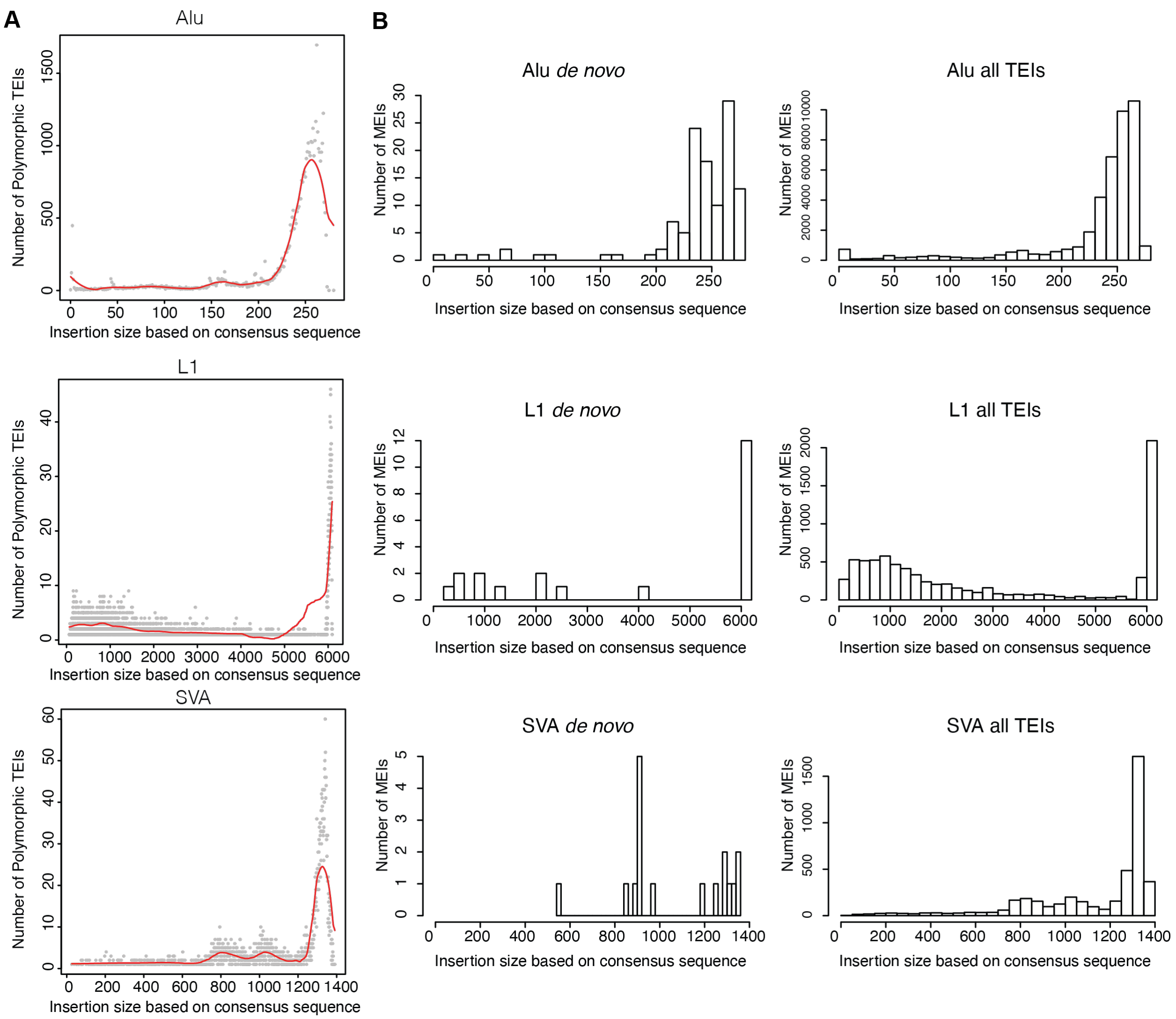

Fig. S10. Estimated insertion size of TEIs. **A** Number of TEIs identified with a certain estimated insertion size for parental, ASD, and control individuals. The red line represents the Loess Regression with a 25% smoothing span. Alu N=42,045, L1 N=7,872, SVA N=4,375. **B** Number of TEIs for each insertion size bin for *de novo* insertions or all insertions including both polymorphic and *de novo*. Since we used the position of clipped reads mapping to a consensus sequence to estimate size, this does not account for variable repeat expansions length or polyA tail variability. All: Alu N=42,045, L1 N=7,872, SVA N=4,375; *de novo:* Alu N=188, L1 N=22, SVA N=17.

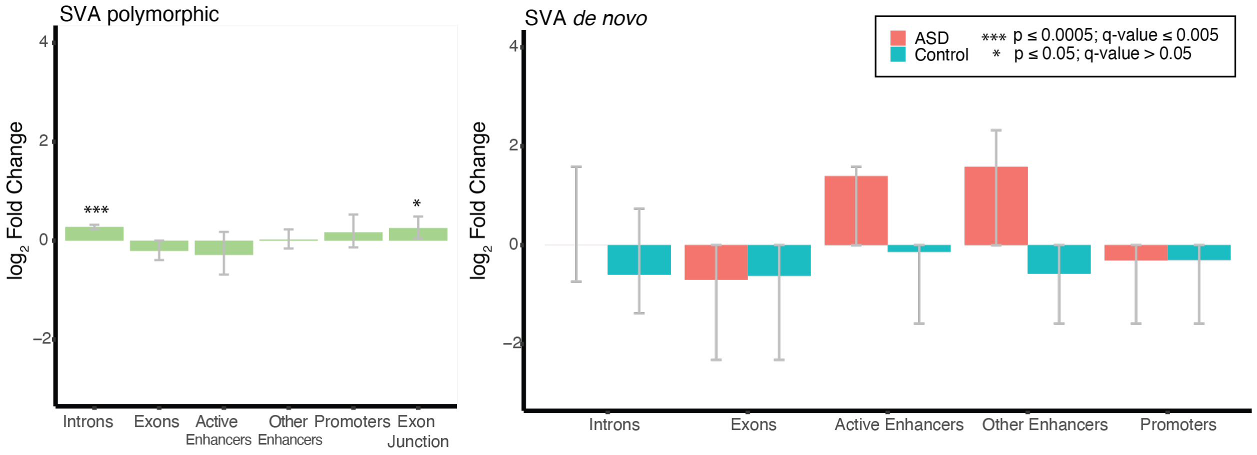

Fig. S11. Enrichment and depletion of TEIs in coding and gene regulatory regions. SVA polymorphic and *de novo* TEIs from parental individuals do not show a depletion in exons and regulatory regions in the developing fetal brain, but the number of insertions in these regions are generally fewer than Alu and L1 TEIs (Additional File 1: Table S6). 10,000 random simulations were performed for both polymorphic and *de novo* TEIs based on the observed rates. Log_2 fold change of observed compared to expected counts in different genomic regions are shown for coding and gene regulatory regions. 95% confidence intervals were estimated based on the empirical distribution of the random simulations. Two-sided empirical p-values and Benjamini–Yekutieli q-values based on multiple correction of all enrichment and depletions performed are represented.

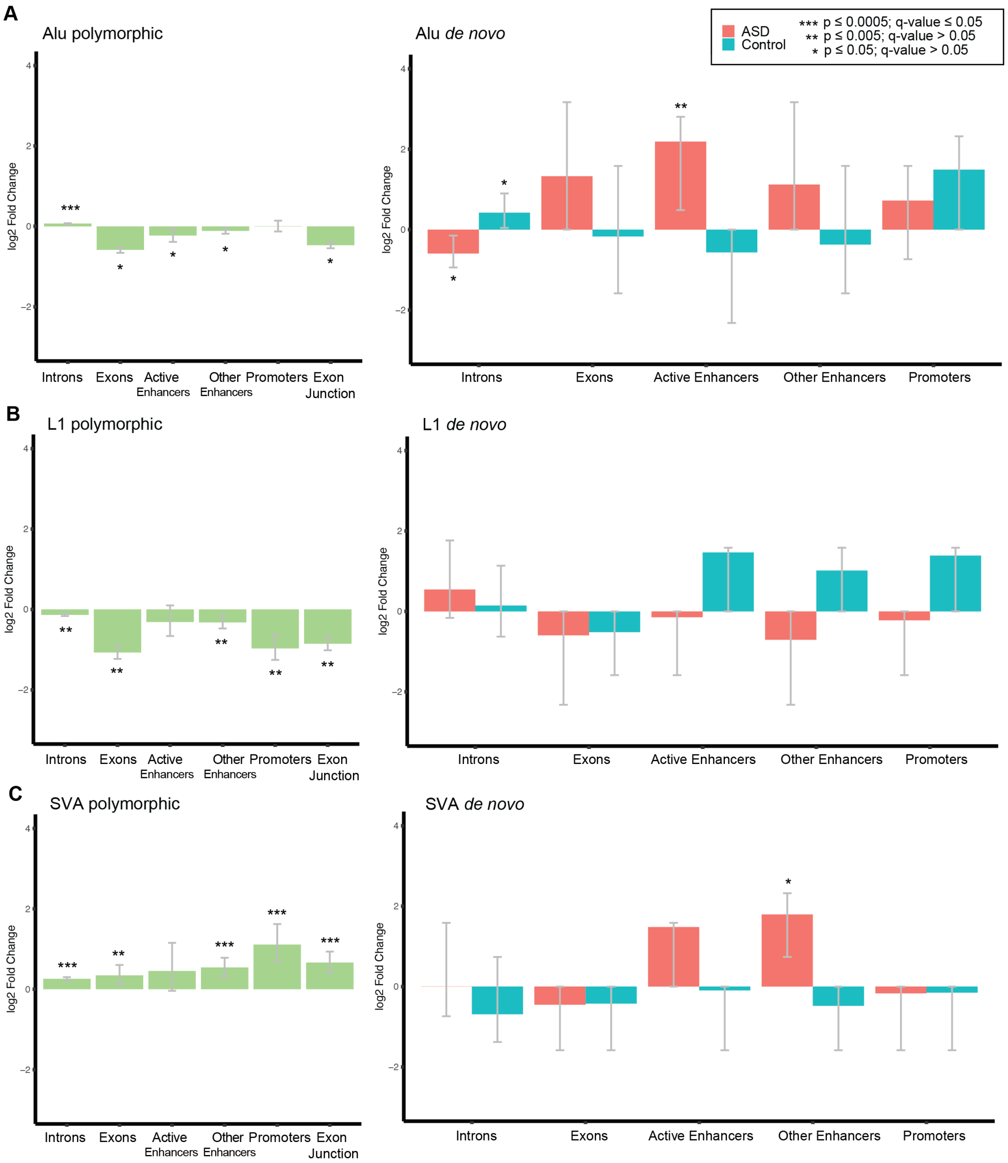

Fig. S12. Enrichment and depletion of TEIs in coding and gene regulatory regions. Log_2 fold change of TEIs identified in the SSC cohort compared to expected counts in different genomic regions are shown for coding and gene regulatory regions. The expected counts were obtained by performing 10,000 random simulations using a position probability matrix of the L1 endonuclease nicking site preference. Similar trends are observed compared to a fully random insertion model for **A** Alu, **B** L1, and **C** SVA polymorphic and de novo TEIs (Fig. 4), although SVA polymorphic insertions show a greater enrichment in this model. Two-sided empirical p-values and Benjamini–Yekutieli q-values are represented.

Supplemental Tables

Table. S1. Polymorphic insertions sample sizes

	L1	Alu	SVA
**ASD Cases**	2,286	2,285	2,287
**Unaffected Siblings**	1,856	1,855	1,858
**Fathers**	2,286	2,285	2,287
**Mothers**	2,286	2,286	2,288

Table. S3. *De novo* insertion rates and sample sizes

	L1	Alu	SVA
**ASD sample size**	2,286	2,286	2,288
**Controls sample size**	1,856	1,857	1,860
**ASD number of *de novo* insertions**	12	62	9
**Controls number of *de novo* insertions**	10	57	8
**ASD *de novo* rates**	0.0052	0.0271	0.0039
**Control *de novo* rates**	0.0054	0.0307	0.0043
**ASD + Control *de novo* insertions**	22	119	17
**ASD + Control sample size**	4142	4143	4148
**ASD + Control *de novo* rates**	0.0053	0.0287	0.0041
**1 in X births**	188.27	34.82	244
**Sensitivity 39.4x HG002**	0.59	0.84	0.80
**Sensitivity heterozygous TEIs with young reference and KNR filters 39.4x HG002**	0.62	0.84	0.84
***De novo* rates adjusted**	0.0085	0.0341	0.0049
**1 in X births adjusted**	117.15	29.36	205.66
**Confidence interval rates lower adjusted**	0.0060	0.0287	0.0030
**Confidence interval rates upper adjusted**	0.0118	0.0400	0.0075
**Confidence interval lower 1 in x adjusted**	167.65	34.79	335.63
**Confidence interval upper 1 in x adjusted**	84.51	24.98	133.58

Table. S4. *De novo* insertions that overlap the top 10% expressed genes in the neocortex during development

	**Early Prenatal**	**Late Prenatal**	**Childhood**	**Adolescence**	**Adulthood**
**Genes with *de novo* Alu insertions in ASD**	CSDE1<br>SYT1<br>KBTBD6<br>TCF25<br>EPS15	CSDE1<br>SYT1<br>TCF25<br>RPH3A<br>EPS15	CSDE1<br>SYT1<br>TCF25<br>RPH3A<br>EPS15	CSDE1<br>SYT1<br>TCF25<br>RPH3A<br>EPS15	CSDE1<br>SYT1<br>TCF25<br>RPH3A<br>EPS15
**Genes with *de novo* Alu insertions in control**	DCLK2<br>SF3A1	DCLK2	DCLK2	DCLK2	
**Genes with *de novo* L1 insertions in ASD**	DAB1				
**Genes with *de novo* L1 insertions in control**	EPHA7				

Overlap of genes with *de novo* insertions and the top 10% expressed genes in neocortex brain regions during development. Early prenatal: 8-19 postconceptional weeks (PCW), Late prenatal: 21-37 PCW, Childhood (4 months -11 years), Adolescence: 13-19 years, and Adulthood: 21-40 years. Genes with *de novo* SVA insertions did not overlap with any of the categories.

Table. S5. Number of *de novo* insertions overlapping regions with epigenetic annotation in fetal brain

Sample and Genomic Region	Alu	L1	SVA
**ASD introns**	18	8	4
**Control introns**	34	5	2
**ASD exons**	4	0	0
**Control Exons**	1	0	0
**ASD active enhancers**	3	0	1
**Control active enhancers**	0	1	0
**ASD other enhancers**	4	0	2
**Control other enhancers**	1	1	0
**ASD promoters**	1	0	0
**Control promoters**	2	1	0

Table. S6. Number of observed polymorphic insertions in parental SSC samples overlapping regions with epigenetic annotation in fetal brain

Genomic Region	Alu	L1	SVA
**Introns**	28,498	4,564	2,727
**Exons**	1,065	140	169
**Active enhancers**	254	44	34
**Other enhancers**	1,560	249	205
**Promoters**	449	42	81
**Exon junctions**	1,031	145	188

Table. S8. Memory and time cost of xTea on different numbers of CPU cores

Cores	Memory	Time
1 core	3,011,224K	30hrs
2 cores	5,395,264K	22hrs
4 cores	7,575,888K	16hrs
8 cores	15,112,408K	7hrs
16 cores	19,256,964K	3hr 50mins

References

1. Lee S, Johnson J, Vitzthum C, Kirli K, Alver BH, Park PJ. Tibanna: software for scalable execution of portable pipelines on the cloud. Bioinformatics. 2019;35(21):4424-6.
2. Quinlan AR, Hall IM. BEDTools: a flexible suite of utilities for comparing genomic features. Bioinformatics. 2010;26(6):841-2.
3. Collins RL, Brand H, Karczewski KJ, Zhao X, Alfoldi J, Francioli LC, et al. A structural variation reference for medical and population genetics. Nature. 2020;581(7809):444-51.
4. Gardner EJ, Lam VK, Harris DN, Chuang NT, Scott EC, Pittard WS, et al. The Mobile Element Locator Tool (MELT): population-scale mobile element discovery and biology. Genome Res. 2017;27(11):1916-29.
5. Chen H. VennDiagram: Generate High-Resolution Venn and Euler Plots. R package version 1.6.19. https://CRAN.R-project.org/package=VennDiagram. 2018.
6. Chu C, Borges-Monroy R, Viswanadham VV, Lee S, Li H, Lee EA, et al. Comprehensive identification of transposable element insertions using multiple sequencing technologies. Nat Commun. 2021.
7. Wang J, Song L, Grover D, Azrak S, Batzer MA, Liang P. dbRIP: a highly integrated database of retrotransposon insertion polymorphisms in humans. Hum Mutat. 2006;27(4):323-9.
8. Beck CR, Collier P, Macfarlane C, Malig M, Kidd JM, Eichler EE, et al. LINE-1 retrotransposition activity in human genomes. Cell. 2010;141(7):1159-70.
9. Ewing AD, Kazazian HH, Jr. High-throughput sequencing reveals extensive variation in human-specific L1 content in individual human genomes. Genome Res. 2010;20(9):1262-70.
10. Ewing AD, Kazazian HH, Jr. Whole-genome resequencing allows detection of many rare LINE-1 insertion alleles in humans. Genome Res. 2011;21(6):985-90.
11. Hormozdiari F, Alkan C, Ventura M, Hajirasouliha I, Malig M, Hach F, et al. Alu repeat discovery and characterization within human genomes. Genome Res. 2011;21(6):840-9.
12. Huang CR, Schneider AM, Lu Y, Niranjan T, Shen P, Robinson MA, et al. Mobile interspersed repeats are major structural variants in the human genome. Cell. 2010;141(7):1171-82.
13. Iskow RC, McCabe MT, Mills RE, Torene S, Pittard WS, Neuwald AF, et al. Natural mutagenesis of human genomes by endogenous retrotransposons. Cell. 2010;141(7):1253-61.
14. Stewart C, Kural D, Stromberg MP, Walker JA, Konkel MK, Stutz AM, et al. A comprehensive map of mobile element insertion polymorphisms in humans. PLoS Genet. 2011;7(8):e1002236.
15. Smit A, Hubley, R & Green, P. . RepeatMasker Open-4.0. <http://www.repeatmasker.org>. 2013-2015.
16. Thorvaldsdottir H, Robinson JT, Mesirov JP. Integrative Genomics Viewer (IGV): high-performance genomics data visualization and exploration. Brief Bioinform. 2013;14(2):178-92.
17. Lee E, Iskow R, Yang L, Gokcumen O, Haseley P, Luquette LJ, 3rd, et al. Landscape of somatic retrotransposition in human cancers. Science. 2012;337(6097):967-71.
18. Zhou W, Emery SB, Flasch DA, Wang Y, Kwan KY, Kidd JM, et al. Identification and characterization of occult human-specific LINE-1 insertions using long-read sequencing technology. Nucleic Acids Res. 2020;48(3):1146-63.
19. Ewing AD, Smits N, Sanchez-Luque FJ, Faivre J, Brennan PM, Richardson SR, et al. Nanopore Sequencing Enables Comprehensive Transposable Element Epigenomic Profiling. Mol Cell. 2020;80(5):915-28 e5.
20. Zook JM, Catoe D, McDaniel J, Vang L, Spies N, Sidow A, et al. Extensive sequencing of seven human genomes to characterize benchmark reference materials. Sci Data. 2016;3:160025.
21. Zook JM, Hansen NF, Olson ND, Chapman L, Mullikin JC, Xiao C, et al. A robust benchmark for detection of germline large deletions and insertions. Nat Biotechnol. 2020.

22. Li H, Feng X, Chu C. The design and construction of reference pangenome graphs with minigraph. Genome Biol. 2020;21(1):265.
23. Robinson JT, Thorvaldsdottir H, Winckler W, Guttman M, Lander ES, Getz G, et al. Integrative genomics viewer. Nat Biotechnol. 2011;29(1):24-6.
24. Karolchik D, Hinrichs AS, Furey TS, Roskin KM, Sugnet CW, Haussler D, et al. The UCSC Table Browser data retrieval tool. Nucleic Acids Res. 2004;32(Database issue):D493-6.
25. Abrahams BS, Arking DE, Campbell DB, Mefford HC, Morrow EM, Weiss LA, et al. SFARI Gene 2.0: a community-driven knowledgebase for the autism spectrum disorders (ASDs). Mol Autism. 2013;4(1):36.
26. Lek M, Karczewski KJ, Minikel EV, Samocha KE, Banks E, Fennell T, et al. Analysis of protein-coding genetic variation in 60,706 humans. Nature. 2016;536(7616):285-91.
27. Roadmap Epigenomics C, Kundaje A, Meuleman W, Ernst J, Bilenky M, Yen A, et al. Integrative analysis of 111 reference human epigenomes. Nature. 2015;518(7539):317-30.
28. Bao W, Kojima KK, Kohany O. Repbase Update, a database of repetitive elements in eukaryotic genomes. Mob DNA. 2015;6:11.
29. Grandi FC, Rosser JM, An W. LINE-1-derived poly(A) microsatellites undergo rapid shortening and create somatic and germline mosaicism in mice. Mol Biol Evol. 2013;30(3):503-12.
30. Hancks DC, Kazazian HH, Jr. SVA retrotransposons: Evolution and genetic instability. Semin Cancer Biol. 2010;20(4):234-45.
31. R Core Team. R: A language and environment for statistical computing. Vienna, Austria: R Foundation for Statistical Computing; 2019.
32. Miller JA, Ding SL, Sunkin SM, Smith KA, Ng L, Szafer A, et al. Transcriptional landscape of the prenatal human brain. Nature. 2014;508(7495):199-206.
33. Hawrylycz MJ, Lein ES, Guillozet-Bongaarts AL, Shen EH, Ng L, Miller JA, et al. An anatomically comprehensive atlas of the adult human brain transcriptome. Nature. 2012;489(7416):391-9.
34. Parikshak NN, Luo R, Zhang A, Won H, Lowe JK, Chandran V, et al. Integrative functional genomic analyses implicate specific molecular pathways and circuits in autism. Cell. 2013;155(5):1008-21.
35. Evrony GD, Cai X, Lee E, Hills LB, Elhosary PC, Lehmann HS, et al. Single-neuron sequencing analysis of L1 retrotransposition and somatic mutation in the human brain. Cell. 2012;151(3):483-96.
36. Untergasser A, Cutcutache I, Koressaar T, Ye J, Faircloth BC, Remm M, et al. Primer3--new capabilities and interfaces. Nucleic Acids Res. 2012;40(15):e115.
37. Kent WJ. BLAT--the BLAST-like alignment tool. Genome Res. 2002;12(4):656-64.
